# Correlates of Health-Protective Behavior During the Initial Days of the COVID-19 Outbreak in Norway

**DOI:** 10.3389/fpsyg.2020.564083

**Published:** 2020-10-06

**Authors:** Janis H. Zickfeld, Thomas W. Schubert, Anders Kuvaas Herting, Jon Grahe, Kate Faasse

**Affiliations:** ^1^Department of Psychology, University of Oslo, Oslo, Norway; ^2^Mannheimer Zentrum für Europäische Sozialforschung, University of Mannheim, Mannheim, Germany; ^3^Department of Psychology, Pacific Lutheran University, Tacoma, WA, United States; ^4^School of Psychology, University of New South Wales, Sydney, NSW, Australia

**Keywords:** COVID-19, coronavirus, health protective behavior, perceived risk, concern, Norway

## Abstract

The coronavirus outbreak manifested in Norway in March 2020. It was met with a combination of mandatory changes (closing of public institutions) and recommended changes (hygiene behavior, physical distancing). It has been emphasized that health-protective behavior such as increased hygiene or physical distancing are able to slow the spread of infections and *flatten the curve*. Drawing on previous health-psychological studies during the outbreak of various pandemics, we investigated psychological and demographic factors predicting the adoption and engagement in health-protective behavior and changes in such behavior, attitudes, and emotions over time. We recruited a non-representative sample of Norwegians (*n* = 8676) during a 15-day period (March 12–26 2020) at the beginning of the COVID-19 outbreak in Norway. Employing both traditional methods and exploratory machine learning, we replicated earlier findings that engagement in health-protective behavior is associated with specific demographic characteristics. Further, we observed that increased media exposure, perceiving measures as effective, and perceiving the outbreak as serious was positively related to engagement in health-protective behavior. We also found indications that hygiene and physical distancing behaviors were related to somewhat different psychological and demographic factors. Over the sampling period, reported engagement in physical distancing increased, while experienced concern or fear declined. Contrary to previous studies, we found no or only small positive predictions by confidence in authorities, knowledge about the outbreak, and perceived individual risk, while all of those variables were rather high. These findings provide guidance for health communications or interventions targeting the adoption of health-protective behaviors in order to diminish the spread of COVID-19.

## Introduction

On the 30th of January 2020, the World Health Organization (WHO) declared the outbreak of a new coronavirus type (SARS-CoV-2) a Public Health Emergency of International Concern. While first cases of COVID-19, caused by this virus, were reported in the Chinese city of Wuhan at the end of December 2019, by the end of March 2020 the virus had spread to all populated continents, resulting in exponential growth and more than 700.000 recorded infections and 30.000 fatalities worldwide. At that time, COVID-19 had already significantly impacted physical and psychological health in many countries, with consequences for many individuals’ daily lives and economic situations.

Increasing evidence about COVID-19 suggests that adopting widespread public behavior change can have strong influences on controlling the virus’ spread and limiting its harmful consequences on physical health and healthcare systems (Ferguson et al., 2020; Li et al., 2020). Some of these changes may be enforced by states (e.g., closure of schools), while others may be advised but not strictly enforced (e.g., reduction of group size in public), and others may be advised but outside of a state’s control (e.g., hand washing in private). Experiences from previous disease outbreaks such as Ebola, SARS, and the swine flu suggests that psychological factors including attitudes and affective reactions have a significant impact on whether individuals adopt health protective behavior or not (e.g., Tang and Wong, 2003; Bish and Michie, 2010; Bults et al., 2011). Facilitating such behavior change during an outbreak is an important task of applied psychology during the COVID-19 outbreak (Lunn et al., 2020; Van Bavel et al., 2020).

In the current study, we explore what demographic and psychological variables predicted the adoption and engagement in health-protective behavior and how attitudes and self-reported behaviors changed over the course of a period of 15 days during the COVID-19 outbreak in Norway. Norway represents an interesting case as it featured the second highest rate of confirmed cases per capita (after Italy) at the beginning of data collection (12th March), while having Europe’s third lowest population density. Four weeks after the closing of schools and beginning of our data collection, Norway had managed to reverse the growth of hospitalizations due to COVID-19. Our data are collected during this period. While our data are cross-sectional rather than longitudinal, they allow the description of a social change process, in addition to exploring correlates of individual behavior.

Protective behavior in a pandemic can be categorized broadly into three types: preventive, avoidant, and management behavior. Preventive behavior includes mainly increase in hygiene (e.g., handwashing), avoidant behavior refers mainly to physical distancing^1^, and management includes taking medication and seeking help from health professionals and use of help lines. An important question to curb infections is, what individual factors predict this kind of behavior. Bish and Michie (2010) reviewed the literature on this following the SARS crisis. They particularly focused on reported associations between demographic factors, attitudes, and behavioral measures (reported, intended, or actual behavior). Most reviewed studies were carried out in the middle of actual outbreaks, mostly of influenza and the SARS coronavirus (SARS-CoV). The review found that preventive and avoidant behavior was predicted by a few demographic factors. These behaviors were more common among women, older people, and people indicating a higher education level (cf. Ibuka et al., 2010; Agüero et al., 2011; Tooher et al., 2013; Moran and Del Valle, 2016; Zettler et al., 2020). More recent evidence has also identified household size as a crucial variable. People living in larger households seem more likely to take precautions (Ibuka et al., 2010), presumably out of increased fear of getting infected or out of increased sense of responsibility for others, or both. The driving factor may be the presence of school-aged children (Agüero et al., 2011; for contradicting findings, see Bults et al., 2011).

Preventive and avoidant behavior were also related to psychological factors. In particular, they were found to increase with perceived susceptibility to the disease (i.e., perceived likelihood of contracting the virus) and perceived severity of symptoms increase (Tang and Wong, 2003; Agüero et al., 2011; Tooher et al., 2013; Gershon et al., 2018; Webster et al., 2020). In a Spanish sample, the adoption of preventive measures during an influenza outbreak was increased by perceived effectiveness of these behaviors in reducing the risk of infection (Agüero et al., 2011; see also Tang and Wong, 2003). These observations are in line with classic and modern versions of expectancy-value theories, where expectancy equals susceptibility and severity equals value. For instance, the Theory of Planned Behavior explains behavior as deriving from intentions that are influenced by attitudes, perceived social norms, and perceived behavioral control or effectiveness of the behavior (Ajzen, 1991). Risk and severity may mediate effects of demographic variables, especially for gender.

Pandemics highlight the interdependence among individuals, and citizens’ relations to their government. Bish and Michie (2010, p. 817) conclude from their review “that having a high level of trust in authorities and satisfaction with the communications received about the disease is associated with compliance with preventive, avoidant, and management behaviors.” Evidence from actual outbreaks confirms this (Liao et al., 2010; Bults et al., 2011).

It seems that little is known on how crucial psychological variables develop during the course of an epidemic. Information on behavioral change over time is important for modeling a pandemic’s course, and providing appropriate health messaging over time (Poletti et al., 2012; Collinson et al., 2015). Over the course of the first wave of the 2009 influenza (H1N1) epidemic in Hong Kong, knowledge on modes of transmission did not improve, the adoption of avoidant behavior did not change, and, surprisingly, physical distancing declined, suggesting that changes might sometimes be counterintuitive (Cowling et al., 2010). This may be due to the ongoing nature of the threat, the requirement to consistently engage in sometimes complex and unpleasant behaviors over a long period of time, and information or media fatigue resulting in reduced behavioral engagement. During the H1N1 outbreak in the Netherlands, perceived severity and anxiety decreased over time in line with better estimates of fatality, but also in line with claims that citizens can be fatigued by media reports (Collinson et al., 2015). It thus seems important to observe the time course of the involved psychological variables.

Wise et al. (2020) surveyed 1591 US-American participants between 3/11/20 and 3/16/20, focusing on perceived risk from the virus and propensity to engage in protective behaviors. Their sample (recruited through Prolific.co) had roughly equal numbers of women and men and a median age of 30, skewing young. Participants saw a medium risk of getting infected themselves (43%), which rose during the time of the sampling. Participants reported that they washed their hands more and stayed at home more than usual, and this also increased during the sampling period. Notably, they were able to reassess a subsample of participants, once at the beginning and once at the end of the sampling period, and confirmed that these changes also occurred within participants.

Self-reported increased hand-washing and physical distancing were predicted by perceived likelihood of becoming infected, but not severity of illness. To a lesser extent, perceived impact from global consequences also predicted both behaviors. Wise et al. (2020) used multiple regressions with 10 different risk perceptions as simultaneous predictors for these analyses and controlled for age. The other predictors (e.g., likelihood of infecting somebody else) did not predict behavior above risk to self, and neither did age. Wise et al. (2020) also identified a subgroup in their sample that perceived low risk and disengaged from information seeking.

In a non-representative sample of 1210 respondents from 194 cities in China during the initial phase of the pandemic, about one third reported moderate to severe anxiety. Interestingly, precautionary measures (e.g., hand hygiene, wearing a mask) were associated with lower levels of stress and anxiety, suggesting successful coping and belief in the behavior’s effectiveness (Wang et al., 2020).

In a small, mostly British, community sample (*n* = 324) collected between March 27th and 28th, experiencing fear was the only positive and stable predictor of health-protective behavior (Harper et al., 2020). Sampling 770 US adolescents from the 20th to the 22nd of March, health-protective behavior including physical distancing and hand washing was positively predicted by perceived severity of the outbreak and social responsibility, as well as negatively predicted by self-interest (Oosterhoff, 2020).

Finally, a nearly identical version of the questionnaire employed in the current project was distributed among Australian adults (*n* = 2174) between the 2nd and 9th of March (Faasse and Newby, 2020). As the number of cases was considerably low in Australia at that time (<100), the authors observed low prevalence of physical distancing behavior but rather high engagement with hygiene behavior. Further, in the study engagement in health-protective behavior was positively predicted by the amount of media exposure, concern or worry about the outbreak, perceived severity of the outbreak, confidence in scientists and health professionals, and accurate knowledge about COVID-19. Perceived likelihood of being infected was not a significant predictor of engagement with health behaviors.

The first infected case in Norway became known on February 26. The number of known infections grew at a relatively slow pace to 227 until March 9, without much action by authorities or concern in the population. The total population of Norway is 5.4 Million. Because authorities had been blindsided by an influx of infected people coming back from winter holidays in Italian and Austrian skiing locations, infections then suddenly increased to 804 until March 12, and community spread was assumed.

That day, on which we started data collection, was tumultuous: The first death was registered. Because Norway lacked testing capacity, a change in testing criteria was announced, prioritizing severely sick people and health personnel rather than travelers. Mildly and moderately ill people did not have access to testing throughout the sampling period. Also on March 12, the Norwegian Institute of Public Health (Folkehelseinstitutet, FHI) published a report predicting that between 20 and 80% of the Norwegian Population would be infected in the first wave, which was expected to take up to 1 year (Folkehelseinstiuttet, 2020). That report was widely publicized. Finally, on the same day the government announced comprehensive measures to fight the virus, most notably shutting down schools and kindergartens, training facilities, and all cultural events. Increased stocking up on food and supplies lead to empty shelves in some grocery shops, which was documented on social media.

On March 13, it was reported that many Norwegians left cities toward holiday homes in remote locations, which led to a rebuke by the authorities due to risk of spreading the virus. Travel by foreigners without residence permits to Norway via plane or boat was shut down March 15. The same day, in an extraordinary announcement, the Norwegian King asked people to stand together and follow the authorities’ advice. On March 16, FHI published a general call for increased physical distancing, and rules about quarantine, assemblies, and visiting cabins started being enforced with fines and short prison sentences. On the 17th, the national TV channel NRK aired a debate in which a medical doctor argued that Norway should go into total isolation and that FHI was too lax. This was seen as controversial, praised by some, but criticized by many others. The number of hospitalizations passed 200 on March 24, with 10 people dead.

Three representative data sets are available with Norwegian samples that help to anchor our data. Sætrevik (2020) reported data from a representative sample of more than 4000 Norwegians between March 20 and March 29. Respondents thought the average Norwegian was likely to be infected by the coronavirus (46% of the panel said “Somewhat high” or “Very high”), while fewer thought that this would happen to themselves. Quite few (8%) believed that they were at risk of becoming seriously ill themselves.

Kantar. no (Gallup) conducted web interviews with representative samples *N* = 947 and *N* = 1538, on March 12–13 and March 19–20, respectively, thus at the beginning and end of our first week of data collection (Kantar, 2020a,b). On March 19/20, the vast majority (>85%) said that they had high or very high confidence that the health authorities would take the necessary measures to handle the situation in the best possible way, and that they provided accurate information on the situation. Both numbers had increased compared to the week before. Less than half, 42% (up from 37 a week earlier), expected that they would likely or very likely be infected (up by 5% during the last week), while 18% said that was unlikely or very unlikely. A large group, 37%, was unsure, saying it was neither likely nor unlikely. Answers judging infection as likely or very likely were more frequent than average among inhabitants of Oslo and people younger than 44, but less frequent among people older than 60 (only 20%). The same age effect was reported by Sætrevik (2020). When asked about behavioral changes in the last 7 days, more than half and up to 88% reported increased hygiene behavior, reduced social contact, and increased purchasing of goods. From March 12–13 to March 19–20, the number of people who were worried or very worried about the consequences for themselves or their families (the question did not specify what kind of consequences) increased by 6–78%.

Opinion. no conducted a daily poll of Norwegians from March 13 to March 21 (Opinion, 2020). N varied between 313 and 819 (Gaute Aas Askheim, 2020, March 23, 2020, personal communication). On every day of that period, more than 60% of polled individuals expressed confidence in the measures taken by the authorities and trust in the information given by them to the public; confidence actually increased from 65% on March 13–75% on March 17, and then fell again slightly.

In the current project we investigated the influence of psychological and demographic variables in predicting health-protective behavior in a Norwegian sample. Simultaneously, we focused on exploring the trajectories and developments of reported behavior, attitudes, and affective reactions during a 15-day period during the outbreak of COVID-19. We focused mainly on two aspects of protective behavior: preventive and avoidance behavior (Bish and Michie, 2010). We did not focus on management behaviors, such as taking medicine or seeing health-professional, as no medication was available at the time of data collection and the main focus was on minimizing transmission and rapid dissemination by flattening the curve through hygiene practices and physical distancing (Ferguson et al., 2020).

Note that our sampling strategy primarily reached participants who were already engaged in discussing topics related to COVID-19, and are presumably more concerned than average. We were thus less likely to sample a lot of participants who viewed the risk as low and were disengaged from seeking information on COVID-19. Our data are thus by no means representative. Absolute means should be interpreted as being at the upper end of the real distribution. Our analyses focus on relations between variables, which we assume to be generalizable to the larger population. Also note that our data are not longitudinal, and we cannot draw causal conclusions. We nevertheless use the term prediction to describe the results of regression analyses for ease of phrasing.

Our analysis strategy in identifying important variables predicting engagement in health-protective behavior was twofold. First, we focused on a theory-driven strategy based on reviews and previous studies relating to health epidemics. Second, we employed an exploratory data-driven machine learning approach in order to classify important variables. Based on reviews concerning factors predicting behavior during pandemics and recent research (e.g., Wise et al., 2020), we derived the following hypotheses for the first strategy:

(I) Engagement in health-protective behavior was expected to be predicted by gender, education level, age, and household size. Women, individuals with higher education level, older individuals, as well as those from larger households were expected to have more engagement in health-protective behavior.
(II) We expected that effects in I were mediated by own perceived risk (likelihood and severity; for gender, education level, and age) or by perceived risk of close others (likelihood and severity; for household size). Females, individuals with higher education or older age should show increased perceived risk, which in turn should be associated with higher reports in health-protective behavior. Similarly, larger household size should be associated with higher perceived risk for close others, which in turn should positively predict health-protective behavior.
(III) Increased confidence and trust in authorities should positively predict engagement in health-protective behavior.

Note that these hypotheses were generated while performing data collection and not completely *a priori*. This was mostly due to time constraints as we wanted to ensure data collection during early periods of COVID-19 outbreak in Norway.

The current project was ethically approved by the Internal Review Board of the University of Oslo. All materials, raw data, and syntaxes are available at our project page: https://osf. io/crs2n/.

## Materials and Methods

### Participants

We recruited a total of 9537 participants residing in Norway through social media (e.g., Facebook, Twitter) and email lists. Data collection took place for 15 days from the 12th of March to the 26th of March 2020. Between March 13 and March 17, we ran a paid ad on Facebook, selecting Norwegian users older than 18 as the target group. The ad reached 33.655 viewers (71% female according to Facebook), of which 1990 clicked through to the survey. The post was shared 165 times and reached over 70.000 Facebook users. The researchers did not themselves share the study in their own networks. After the ad campaign on Facebook ended, the survey was shared on the website of the Department of Psychology (PSI) of the University of Oslo (UiO) and in the Facebook feeds of both PSI and UiO.

**FIGURE 1:**
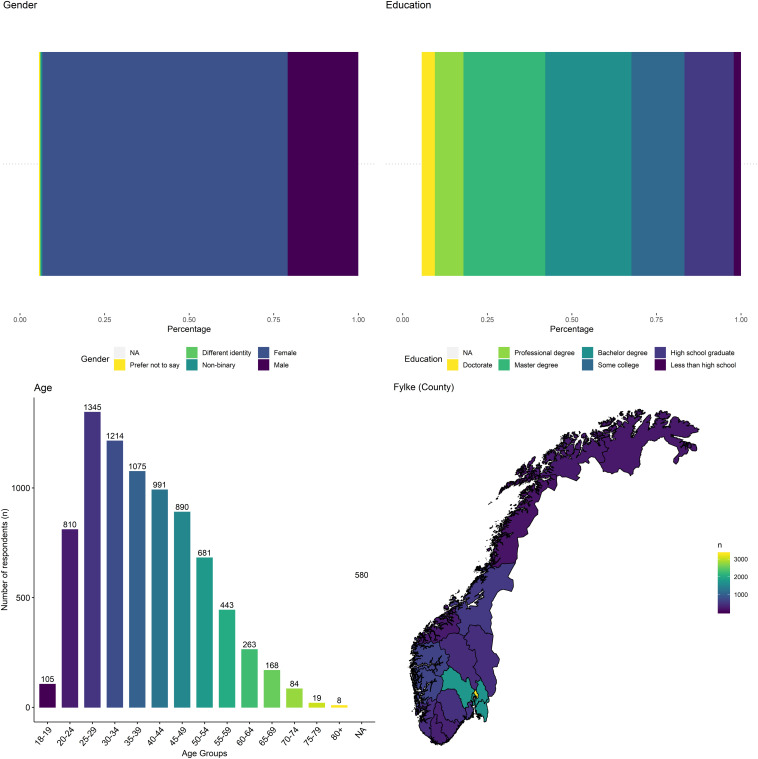
Overview of sample characteristics based on gender, education, age, and residence.

After excluding participants who failed an attention check or spent less than 1 min taking the questionnaire, we arrived at a final sample size of 8676 (6292 females, 1811 males, 59 non-binary or different identity, 28 preferred not to say, 486 missing). The majority of participants were between 20 and 59 years of age. Median age for both male and female participants was between 35 and 39. The majority of participants reported residing in Oslo county (*n* = 3302, 38.1%), while the fewest participants were from Nordland (*n* = 183, 2.1%). Similarly, the majority indicated residing in a large city (*n* = 4299, 49.5%), while the lowest amount came from a rural area (*n* = 938, 10.8%). The majority of the sample indicated a high degree of school education, having earned a college degree (41.8%), whereas a smaller proportion indicated their highest education as less than high school or high school graduate (16.6%). An overview of sample characteristics is provided in Figure 1 or in the Supplementary Materials^2^.

Participants were able to take part in a raffle getting the chance of winning one out of 20 vouchers at a value of 200 NOK. This served mainly to jumpstart the participation; we did not anticipate the large sample ultimately achieved. To participate in the voucher draw, participants were invited to enter their email in a separate follow-up survey that was not linked to the main dataset.

### Materials and Procedure

The main procedure was based on a similar survey conducted in Australia and the US (Faasse and Newby, 2020).

#### Geographical Information

After providing informed consent, we checked whether potential respondents were residing in Norway. Those who did not were thanked and the survey was terminated. We then collected information on participants’ postcodes and the county they resided in. Based on the postcode data, we identified the municipality participants resided in. Using these data, we added the amount of COVID-19 cases for that given municipality on the day the respondent completed the survey based on numbers provided by the Norwegian institute of health [Folkehelseinstitutt (FHI)] and made accessible by the newspaper VG^3^. Similarly, we added information on population density per municipality level based on data from the Statistisk Sentralbyrå.

#### Information Sources

In order to assess the variety and amount of media exposure, we asked participants how much they had seen, read, or heard about COVID-19 [from nothing at all (1) to a lot (4)], how much they think they know about COVID-19 [nothing at all (1) to a lot (4)], and how closely they had been following news about the recent outbreak [from not at all (0) to very closely (10)]. These three items were combined into a mean media exposure score (α = 0.68).

Afterward, we instructed respondents to check all possible sources through which they had been getting information about the COVID-19 outbreak [including news media, social media, official government websites, family member(s), colleague(s) or friend(s), none of the above, and other]. Similarly, we asked participants which out of several sources they trusted the most concerning the outbreak [my doctor, my local hospital, Folkehelseinstitutt, (Norwegian) media, WHO, Norwegian government, state department of health, none of the above, other]. To further investigate respondent’s confidence, we asked how much confidence they had in different sources: the Norwegian government providing full and accurate information, the Chinese government providing full and accurate information, and scientists and medical experts understanding the outbreak. All items were completed on a 10-point scale (not at all confident to very confident, and don’t understand at all to understand very clearly).

Respondents were also asked about how confident they thought health authorities, and hospitals and medical services were able to manage the COVID-19 outbreak [from not at all confident (0) to very confident (100)]. The four items (excluding the item on the Chinese government) were averaged into a confidence score (α = 0.73). The item focusing on the Chinese government was excluded as we mainly intended to focus on confidence in Norwegian health authorities. Additional analyses including the item are presented in the Supplementary Materials.

#### Perceived Risk

Respondents were asked how concerned or worried they were about the COVID-19 outbreak [not at all concerned (1) to extremely concerned (5)]. Participants indicated how likely they thought it would be that they themselves would get infected by COVID-19 and also how likely they thought it would be that close others (family/friends) would get infected [not at all likely (0) to extremely likely (100)]. Similarly, we asked how much participants thought they could do to protect themselves [effectiveness of behavior, I can’t do anything to protect myself (0) to I can do a lot to protect myself (100)]. Asking about perceived severity, participants reported how serious they thought their symptoms would be if they got infected, and what the worst possible outcome could be for a family member or close friend that got the virus [no symptoms (1) to severe symptoms leading to death (6)]. Then, we asked whether participants had already wondered at some point whether they were infected [not at all (0) to very much so (100)]. Finally, we asked respondents whether they thought that too much fuss was being made about the risks of COVID-19 [strongly disagree (1) to strongly agree (5)]. This item was used previously to tap skepticism about warnings in public health crises (Rubin et al., 2015).

#### Emotional States

Affective reactions were captured with several items. Participants reported whether they felt fearful, frightened, anxious, optimistic, encouraged, hopeful, relaxed, furious, outraged, depressed, and sad [strongly disagree (1) to strongly agree (5)]^4^. We averaged the first three items to create a fear score (α = 0.85), items four to six to create a hope score (α = 0.69), items eight and nine to create an anger score (α = 0.80), and the last two items to create a sadness score (α = 0.61). We included items on specific *basic* negative emotions (Ekman, 1992; Ahorsu et al., 2020) and future-oriented positive emotions (Fredrickson, 2001) that we expected to occur commonly in response to health epidemics (Kleinberg et al., 2020; though see Fiske, 2019 for a critique of this approach).

#### Knowledge

In order to test participant’s knowledge about the COVID-19 outbreak, we first asked them to judge whether 16 statements about the virus and disease were true (answer alternatives true, false, and unsure). We then asked participants to indicate what the most common symptoms of COVID-19 were from a list of seven possible symptoms (fever, cough, sore throat, shortness of breath, nausea, vomiting, diarrhea)^5^. Afterward, respondents were prompted to indicate how COVID-19 could spread, according to their knowledge (by air, by water, by mosquitoes, droplets spread through coughing or sneezing, touching surfaces that have been recently touched by someone who is sick, and touching or shaking hands with a person who is sick). The symptoms and transmission items used the same scale (yes, no, unsure). Because the employment of face masks has been a popular debate, we asked who should be wearing a face mask to minimize transmission (healthy people - to prevent infection, sick people - to stop them spreading the virus, everyone, and no one)^6^. Finally, we asked participants to estimate what percentage of people who had been infected with COVID-19 had died from the virus. Respondents were able to provide an answer between 0 and 100%. Out of all correct answers^7^ we constructed a knowledge sum score (ranging from 0 to 31). For the last item, we took the range between 1 and 5% as a correct answer, as official indications had been varying somewhat during the period the study was conducted.

#### Avoidance Behaviors

We then asked respondents to indicate whether they performed 24 different health-protective behaviors in response to the COVID-19 outbreak during the past 2 weeks. These behaviors consisted of physical distancing behavior (13 items, e.g., reduced or avoided going to work or university), hygiene behavior (6 items, e.g., used sanitizing hand gel to clean your hands more often than usual), prosocial behavior (3 items, e.g., helped buying groceries and supplies for people who are in quarantine), and two additional items (e.g., worn a face mask when going out in public)^8^. Responses could be made using four alternatives (yes, no, unsure, not applicable). For each type of behavior, we computed a sum score based on whether the behavior was performed or not. In addition, we computed an overall health/communal-protective behavior sum score based on the physical distancing, hygiene, and prosocial items (summing up all items). Finally, respondents were able to write down whether they did anything else in response to the COVID-19 outbreak.

#### Demographics and Health-Related Information

We collected several items on demographic information and health-related behavior and characteristics. First, participants were asked how likely they would be to get vaccinated in case an effective vaccine for COVID-19 had been developed [would definitely get the vaccine (1) to would definitely NOT get the vaccine (5)]. We then asked to what age groups respondents belonged to (e.g., 18–19, 20–24, 25–29, 30–34, …, 80+) and with what gender they identified (male, female, non-binary, different identity, prefer not to say). Participants then indicated how many children they had (none, 1, 2, more than 2) and the level of their highest education (less than high school, high school graduate, some college, BA degree, MA degree, professional degree, doctorate). We then asked what type of community they lived in (large city, suburb, small city/town, rural area) and how many people (including them) lived in their household (from 1 to 5 or more). Participants then completed some items about their health status, including how they would rate their health in general [poor (1) to excellent (5)], whether they had a flu vaccine within the last year (yes, no, unsure), whether they had been in an affected area with high transmission within the past 2 weeks, whether they had been in close contact with people who are suspected to be infected, whether they had experienced any COVID-19 symptoms, whether close others experienced any symptoms (on all yes, no, unsure), whether they had any chronic health problems that increased their risk, and whether close others had any chronic health problems (both items yes, no, unsure, prefer not to say). Finally, participants were thanked and provided with several links to websites from official sources (WHO, ECDC, FHI) that provided information about the COVID-19 outbreak.

#### Data Analysis

When analyzing data using null hypothesis significance testing (NHST), we set our alpha level at *p* < 0.001. This decision was based on the fact that we employed a considerably large dataset and our findings might have important health-psychological implications (see Lakens et al., 2018). As even small effects will reach statistical significance given large samples, we primarily focus on interpreting effect sizes and their direction and magnitude.

As said above, our analytic strategy was twofold: first a theory-driven step and second an exploratory data-driven machine learning step. For the theory-driven step, we used regular linear regression. The mediation models also tested in this first step employed a bootstrapping method (*n* = 1000) to calculate confidence intervals around the indirect effect.

For the data-driven step, the goal was to classify what variables predicted health-protective behavior out of all predictors we had available in a bottom-up fashion. To do so, we combined supervised machine learning with a partially confirmatory approach (split-half validation) as employed in previous research dealing with large numbers of predictors (e.g., IJzerman et al., 2018). As a supervised machine learning technique, we used conditional random forests, a bootstrap-like algorithm that assesses the relative contribution of each variable on the dependent variable (the signal), therefore being considered a supervised approach (Breiman, 2001). As the name suggests, the algorithm “plants a forest consisting of several trees” that represent the importance of a predictor randomly sampled from the dataset. This procedure is based on out of bag estimates, also called bagging, that features repeated sampling from the original data. In essence, the technique bootstraps several non-parametric regression models and summarizes the importance of each predictor by aggregating and weighting the predictors into a parsimonious set (see Breiman, 2001; IJzerman et al., 2016; Yarkoni and Westfall, 2016). As summarized by IJzerman et al. (2018), employing a supervised machine learning algorithm has several advantages in comparison to classical regression models, and especially using them for exploratory analyses. The algorithm is naive to non-linear relationships, does not assume the direction of a relationship, has less problems with multicollinearity, and has the advantage of assessing each predictors individual role, but also its multivariate interactions with other variables (Strobl et al., 2008).

For our analyses we employed R (Version 3.6.2) and several packages including: dplyr (Wickham and François, 2020), car (Fox et al., 2020), sjmisc (Lüdecke et al., 2020), tidyr (Wickham and Henry, 2020), and stringr (Wickham, 2019) for data recode and wrangling routines, ggpubr (Kassambara, 2020), sp (Pebesma et al., 2020), viridis (Garnier et al., 2018), cowplot (Wilke, 2019), fhidata (White, 2019) for plotting, apaTables (Stanley, 2018) for tables, lavaan (Rosseel et al., 2019) for mediation analyses, and randomForest (Breiman et al., 2018), party (Hothorn et al., 2020), tree (Ripley, 2019), lattice (Sarkar, 2008) for the machine learning analyses.

## Results

### Descriptives

Considering respondents’ information sources, the majority indicated that they received their information about the COVID-19 outbreak from several different sources – on average, participants indicated *M* = 3.15 different sources (*SD* = 1.21). A total of 95% reported news media as an information source, with a smaller number using official government websites (83%) or social media (63%). Less than half of all participants indicated that they used colleagues (42%) or family members (31%) as an information source. No participant reported relying on no source at all.

The majority of participants expressed trust in advice and information from the Norwegian health institute (FHI; 88%). This trust was much smaller for the Norwegian department of health (38%), the Norwegian government in general (34%), and the European Centre for Disease Prevention and Control (ECDC; 33%). A total of 20% of respondents reported trusting (Norwegian) media, and the overall lowest trust was indicated for one’s doctor or general practitioner (10%), and one’s local hospital (12%).

Respondents rated their own perceived likelihood of catching COVID-19 on average somewhat over the midpoint of the 100-point scale (*M* = 60.34, *SD* = 22.27). Assuming they would get infected, the majority predicted to have mild or moderate symptoms (82.7%), while a small proportion reported to expect no (0.4%) or more severe symptoms (14.2%). Participants saw it as even more likely that someone from their family or a close friend would get infected (*M* = 72.85, *SD* = 22.53). When imagining the worst possible outcome for a family member or friend who would get infected, the majority (70.9%) also foresaw potentially worse outcomes including severe symptoms or severe symptoms leading to hospitalization or death. Participants reported that they had already wondered whether they were infected somewhat lower than the midpoint of the scale (*M* = 43.59, *SD* = 35.97). Finally, on average respondents tended to disagree that too much fuss was being made about the risks of the COVID-19 outbreak (*M* = 1.84, *SD* = 1.12 on a 1–5 scale), with only around 10.9% tending to agree or strongly agree.

On average, participants indicated that they were moderately concerned or worried about the outbreak (*M* = 3.09, *SD* = 0.91), with 32.4% being very or extremely concerned. Similarly, on average respondents reported to show the highest levels of fear (*M* = 3.61, *SD* = 1.00), followed by sadness (*M* = 3.11, *SD* = 1.05), hope (*M* = 2.46, *SD* = 0.88), and anger (*M* = 2.26, *SD* = 1.16).

**FIGURE 2:**
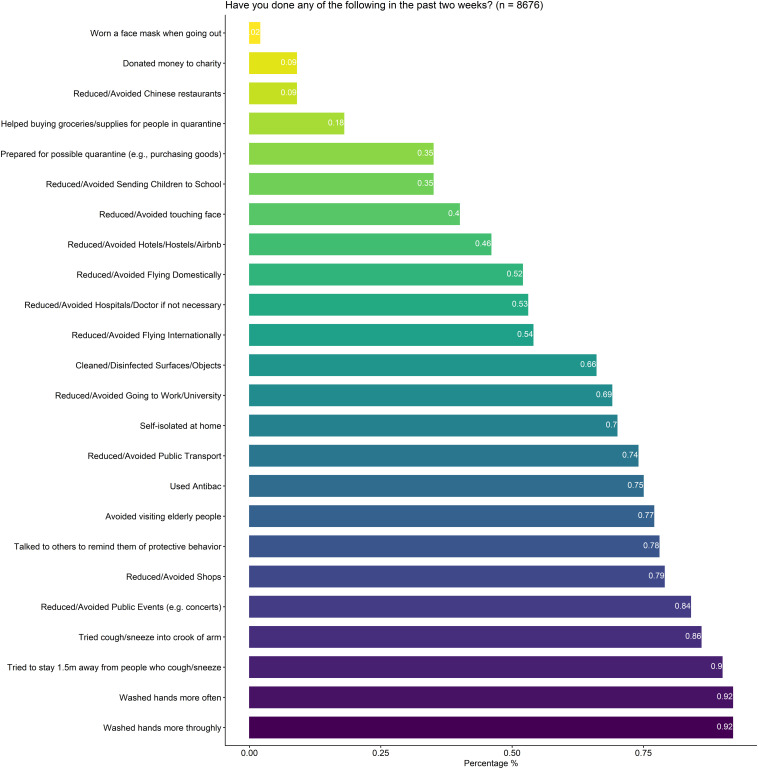
Overview of health/communal-protective behaviors and the percentage of respondents that reported to engage in these.

Considering behavior responses, the majority of respondents reported that they had reduced or avoided going to public events (84%), taking public transport (74%) or going to shops (79%). Similarly, a high percentage of participants disclosed that they had washed their hands more often (92%) and more thoroughly (92%), tried to stay away more than 1 m from others coughing or sneezing (90%), as well as tried to sneeze into the crook of their arm (86%). For prosocial behavior, a majority of respondents indicated that they talked to others and tried reminding them of protective behavior (76%). A rather low occurrence of participants reported that they had avoided Chinese restaurants or neighborhoods specifically (9%) or donated money to charity focusing on combating the COVID-19 outbreak (9%). An overview of all behaviors is provided in Figure 2.

### Factors Predicting Health-Protective Behavior

In order to classify important variables predicting engagement in health-protective behavior we employed two different strategies: a highly confirmatory theory-driven strategy based on reviews and previous studies on the COVID-19 outbreak, and a highly exploratory data-driven approach using a supervised machine learning procedure combined with split-half validation.

#### Theory Driven Approach

**FIGURE 3:**
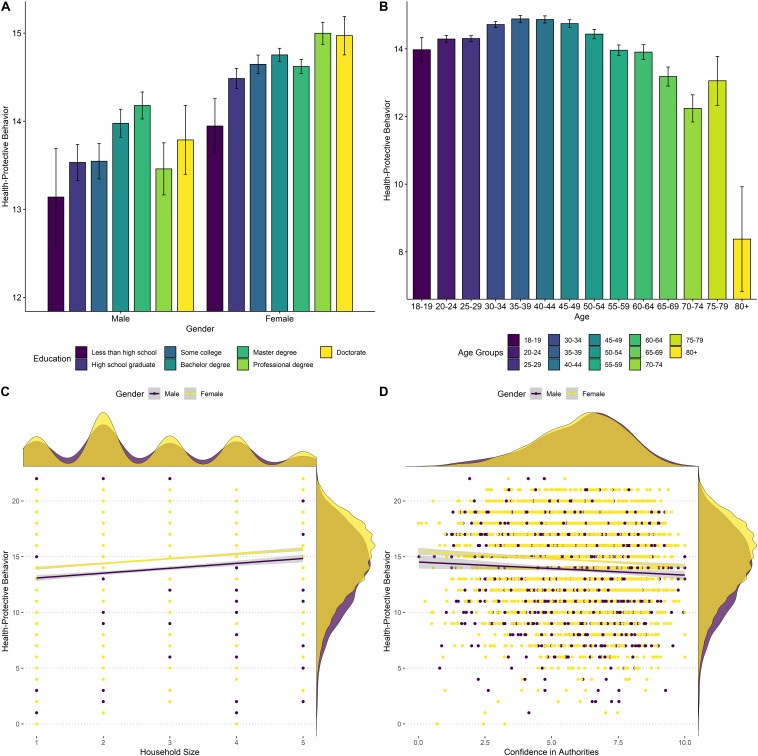
**(A)** Health-protective behavior by gender and education level. **(B)** Health-protective behavior by age group. **(C)** Association of health-protective behavior and household size separately for gender. **(D)** Association between health-protective behavior and confidence in authorities separately for gender.

In order to test hypothesis I, we conducted a linear regression using the health-protective behavior sum score as the outcome and gender, education, age, and household size as predictors (see Table 1 and Figure 3 for results). As predicted, reporting one’s gender as female, indicating a higher education level, as well as a bigger household was associated with significantly more engagement in health-protective behavior. Contrary to our prediction, age showed a negative association with health-protective behavior. However, when inspecting the relationship between age and engagement in health-protective behavior, we observed a non-linear relationship showing first an increase in behavior with increasing age that leveled off at around 40–44 years of age (Figure 3B). Notably, our sample included few individuals over the age of 70, suggesting that these findings should be interpreted with caution. Similarly, when repeating the model with time as a covariate the age effect was not significant, while the other predictors still showed positive effects (see Supplementary Material).

**TABLE 1:** Regression models results using health-protective behavior as the criterion.

**Predictor**	***b***	***b* 95% CI [LL, UL]**	***beta***	***beta* 95% CI [LL, UL]**	***r***
Model 1. Fit: *R*^2^ = 0.040*, 95% CI [0.03,0.05]					
(Intercept)	10.91*	[9.87, 11.96]			
Gender	0.86*	[0.69, 1.03]	0.11	[0.09, 0.13]	0.11*
Age	−0.05*	[−0.08, −0.02]	−0.04	[−0.06, −0.02]	−0.05*
Education Level	0.10*	[0.05, 0.15]	0.04	[0.02, 0.06]	0.04*
Household Size	0.42*	[0.36, 0.47]	0.15	[0.13, 0.18]	0.16*
Model 2. Fit: *R*^2^ = 0.004*, 95% CI [0.00,0.01]					
(Intercept)	15.27*	[14.99, 15.54]			
Confidence in Authorities	−0.13*	[−0.17, −0.09]	−0.06	[−0.09, −0.04]	−0.06*

Gender was dummy coded (0 = Male; 1 = Female). A significant *b*-weight indicates the beta-weight and semi-partial correlation are also significant. *b* represents unstandardized regression weights. *beta* indicates the standardized regression weights. *r* represents the zero-order correlation. *LL* and *UL* indicate the lower and upper limits of a confidence interval, respectively. *Indicates *p* < 0.001.

To test hypothesis III, we regressed the confidence score (mean score based on ratings of confidence in Norwegian government, scientists, health authorities, and medical services) on engagement in health-protective behavior (Table 1 and Figure 3D). Contrary to our prediction, we observed a small negative association. The more confidence respondents expressed in authorities, the less health-protective behavior they reported.

#### Mediation Analyses

We tested four mediation models. The tests are documented in detail in the Supplementary Material (Supplementary Figure S1). In all models, health-protective behavior was the dependent variable. The first three models tested separately whether the effects of gender, age, and education level, respectively, were mediated by two mediators, likelihood and severity of perceived risk. The fourth model tested whether the effect of household size on health-protective behavior was mediated by likelihood and severity of risk to close others.

In short, we found some evidence for mediation of the demographic variables gender, age and education level through likelihood and severity of risk, confirming classic notions of expectancy × value theories. This was especially true for age and education, and the mediation through likelihood. However, all observed mediations were small and partial, and the patterns varied between the different models. This suggests that the demographic variables impact behavior through other channels that were not captured by our measured constructs. We thus do not go into further detail on these here; see the Supplementary Material for further information.

#### Data Driven Approach

Following the strategy laid out above, we employed a data driven approach to identify the strongest predictors that parsimoniously predict health-protective behavior from all predictors we had available. For this purpose, we first split the dataset randomly in half and performed conditional random forests on one half, the training dataset (*n* = 4338). For reproducibility, we actually performed the algorithm using two different seeds and two versions of the amount of variables sampled at each tree (MTry, the square root of the number of variables, 5 or 6). The Spearman Rank correlation among the replications was between 0.96 and 0.98 and therefore considered as stable. According to this analysis of the training dataset, health-protective behavior was best predicted by (in order; see also Supplementary Figure S2):

concern/worry, fear, household size, thinking that too much fuss is made, number of children, perceived effectiveness of behavior, media exposure, sadness, anger, age, relaxation, symptoms (close others), symptoms, perceived risk (likelihood), being to an area with a high number of cases, gender, contact to other individuals showing symptoms, community type, education level, perceived health, knowledge, perceived risk (severity), population density at municipality level.

We observed no evidence that perceived risk (severity) of close others, feeling hope, perceived risk (likelihood) of close others, amount of media sources consumed, actual number of cases per municipality, confidence in authorities and scientists, or taking a flu vaccine within the last year predicted better than random noise.

We then continued to run a regression analysis on health-protective behavior using the second half of the data, the test dataset (*n* = 4338) with the predictors found in training dataset. This was done to reduce random noise from the first step. An overview of the results is provided in Table 2. Health-protective behavior was positively and significantly (at the 0.001 level) predicted by household size, number of children, perceived effectiveness of the behavior, and media exposure, when controlling for all other variables. Similarly, we observed that thinking that people made a fuss about the outbreak and reported age showed significant negative predictions when controlling for the other predictors.

**TABLE 2:** Regression results using health-protective behavior as the criterion.

**Predictor**	***b***	***b* 95% CI[LL, UL]**	***beta***	***beta* 95% CI[LL, UL]**	***r***
Model 3. *R*^2^ = 0.173*, 95% CI[0.14,0.19]					
(Intercept)	7.91*	[5.20, 10.62]			
Concern/Worry	0.27	[0.07, 0.48]	0.07	[0.02, 0.13]	0.26
Fear	0.23	[0.04, 0.42]	0.07	[0.01, 0.13]	0.25
Household Size	0.30*	[0.15, 0.44]	0.11	[0.06, 0.16]	0.18
Fuss	−0.27*	[−0.40, −0.14]	−0.09	[−0.13, −0.05]	−0.20
# Children	0.37*	[0.17, 0.56]	0.12	[0.06, 0.19]	0.11
Perceived Effectiveness	0.02*	[0.01, 0.03]	0.11	[0.07, 0.15]	0.10
Media Exposure	0.33*	[0.14, 0.52]	0.07	[0.03, 0.11]	0.17
Sadness	0.04	[−0.11, 0.20]	0.01	[−0.03, 0.06]	0.15
Anger	0.11	[−0.02, 0.24]	0.04	[−0.01, 0.08]	0.12
Age	−0.16*	[−0.23, −0.08]	−0.12	[−0.17, −0.06]	−0.09
Relaxation	−0.21	[−0.34, −0.07]	−0.07	[−0.12, −0.02]	−0.22
Symptom Close Others	0.11	[−0.20, 0.41]	0.02	[−0.03, 0.06]	0.11
Symptoms	0.48	[0.15, 0.81]	0.07	[0.02, 0.11]	0.15
Perceived Risk (Likelihood)	0.01	[−0.00, 0.01]	0.04	[−0.01, 0.08]	0.14
Area	0.14	[−0.19, 0.48]	0.02	[−0.02, 0.06]	0.03
Gender	0.40	[0.08, 0.72]	0.05	[0.01, 0.09]	0.12
Contact	0.44	[0.06, 0.83]	0.05	[0.01, 0.09]	0.09
Community Type	−0.14	[−0.30, 0.03]	−0.04	[−0.10, 0.01]	−0.03
Education	0.09	[−0.01, 0.19]	0.04	[−0.00, 0.08]	0.05
Perceived Health	−0.01	[−0.17, 0.14]	−0.00	[−0.05, 0.04]	−0.03
Knowledge	−0.05	[−0.10, −0.00]	−0.04	[−0.08, −0.00]	−0.00
Perceived Risk (Severity)	0.06	[−0.13, 0.26]	0.01	[−0.03, 0.06]	0.04
Population Density (Municipality)	0.00	[−0.00, 0.00]	0.01	[−0.05, 0.06]	0.01

A significant *b*-weight indicates the beta-weight are also significant. *b* represents unstandardized regression weights. *beta* indicates the standardized regression weights. *r* represents the zero-order correlation. *LL* and *UL* indicate the lower and upper limits of a confidence interval, respectively. *Indicates *p* < 0.001.

As mentioned earlier, the negative finding concerning age should be interpreted with caution since we sampled a small number of older adults exceeding 70 years of age and considering the relationship between age and health-protective behavior showed a non-linear association, resembling a reverse u-shaped curve. While other variables such as concern or fear showed the strongest variable importance in the first step, they did not emerge as significant predictors from the second step. However, they still showed a similar positive effect as for example media exposure and medium zero-order correlations. The same was true for symptoms and relaxation, with the latter showing a negative prediction.

We repeated the procedure of training machine learning and test using linear regression for hygiene and physical distancing behavior separately. Results differed only minimally and can be found in the Supplementary Materials. For physical distancing, perceived effectiveness of the behavior and respondent’s symptoms had a stronger variable importance. For hygiene behavior, the amount of media sources they were exposed to and whether respondents received a flu vaccine within the last year were more important. Physical distancing was positively and significantly predicted by household size, number of children, whether the respondent experienced symptoms, and perceived effectiveness of one’s own behavior. On the other hand, thinking that people made a fuss and age predicted physical distancing negatively. For hygiene behavior, concern/worry, fear, and media exposure showed a significant positive association when controlling for the other variables. Thinking that ‘too much fuss’ was being made about the risk of COVID-19 predicted hygiene behavior negatively. In general, it seemed that being surrounded with more people, and regarding staying away from others as effective, predicted physical distancing, whereas emotional reactions and media exposure were more important for in engaging in hygiene behavior.

### Development of Behavior, Attitudes, and Affective Reactions Over Time

Finally, we explored the development of behaviors, attitudes, and affective reactions over time. We focused specifically on physical distancing and hygiene behavior (behavior), confidence in authorities, perceived risk likelihood, perceived risk severity (attitudes), and concern/worry, fear, and hope (affective reactions). We regressed each variable on day and day squared. We excluded dates that included less than 50 participants, which was true for the beginning (March 12, *n* = 18) and end of data collection (March 26, *n* = 20). The first day of the time series was thus coded as 1. Thus, we focused on 13 data points per variable (*n* = 8638). Notably, we did not employ a repeated measurement design. We can thus only model changes between participants, but not within, and changes observed over time could be due partially to changes in the sample composition. In order to control for changes in demographics per day we computed four logistic regression models regressing age, gender, education level, and household size on day and day squared. We only observed statistically significant effects for age showing a negative linear effect (*B* = −0.69, *SE* = 0.05) and a positive quadratic effect (*B* = 0.04, *SE* = 0.003), suggesting that the sample in general became younger over time, but then increased in age at the end of the sampling period. As previous analyses suggested that age predicted health-protective behavior and other variables, we added age as a covariate to all models in order to control for it.

**FIGURE 4:**
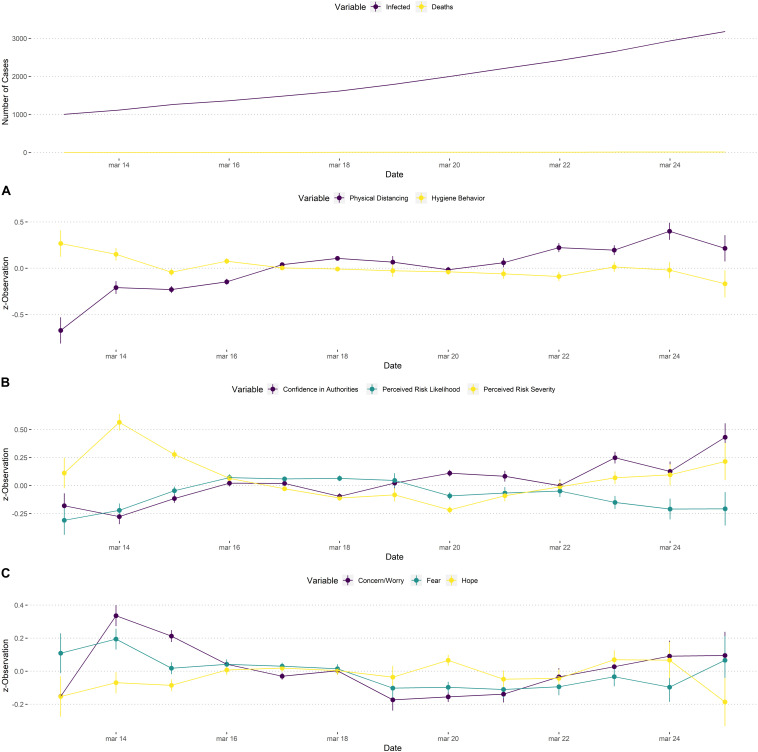
Time series of behavior **(A)**, attitudes **(B)**, and affective reactions **(C)** from the 13th until the 25th of March. All variables are z-standardized to ease comparisons. Error bars represent standard errors. The first row shows number of total infected cases and deaths per day based on data by the FHI.

Results are provided in Table 3 and time series can be found in Figure 4. For behavior, we observed that physical distancing showed a significant positive linear trend. Overall, engagement in physical distancing behavior increased during the days of data collection. On the other hand, hygiene behavior showed no significant linear or quadratic effect. Instead, it showed a small decrease during the first days, but remained rather stable.

**TABLE 3:** Changes in main variables over the sampling period detailed through regressing them on day of answering (linear and quadratic), controlling for participant age.

**Predictors**	***B***	***SE***	***t***
**Physical Distancing**			
Intercept	7.34	0.20	37.22*
Time	0.28	0.06	5.05*
Time^2^	−0.01	0.004	−3.06
Age	−0.05	0.01	−4.35*
**Hygiene Behavior**			
Intercept	5.40	0.09	57.14*
Time	−0.06	0.03	−2.13
Time^2^	0.003	0.002	1.50
Age	−0.01	0.006	−1.74
**Confidence in Authorities**			
Intercept	5.57	0.13	44.10*
Time	0.08	0.04	2.08
Time^2^	−0.002	0.003	−0.65
Age	0.03	0.007	3.68*
**Perceived Risk Likelihood**			
Intercept	58.76	1.71	34.46*
Time	1.97	0.49	4.04*
Time^2^	−0.17	0.04	−4.90*
Age	−0.54	0.10	−5.39*
**Perceived Risk Severity**			
Intercept	2.72	0.06	45.81*
Time	−0.14	0.02	−8.31*
Time^2^	0.01	0.001	7.34*
Age	0.10	0.003	29.83*
**Concern/Worry**			
Intercept	3.41	0.07	48.83*
Time	−0.13	0.02	−6.37*
Time^2^	0.008	0.001	5.45*
Age	0.02	0.004	5.22*
**Fear**			
Intercept	4.06	0.08	52.29*
Time	−0.08	0.02	−3.69*
Time^2^	0.004	0.002	2.66
Age	−0.03	0.005	−6.12*
**Hope**			
Intercept	2.18	0.07	31.71*
Time	0.06	0.02	2.96
Time^2^	−0.004	0.001	−2.59
Age	0.02	0.004	4.27*

*Indicates *p* < 0.001.

Considering attitudes, we observed that confidence in authorities slightly increased during the testing period, though this effect was not statistically significant. Perceived likelihood of catching COVID-19 showed both a significant positive linear trend and a significant negative quadratic trend, first increasing, but later showing a small decrease. Severity of the disease combined a significant negative linear and a positive quadratic trend, first decreasing and then increasing. Taken together, it seems that the more likely catching COVID-19 was reported to be, the less severe respondents estimated it to be over time. Finally, experiencing concern or worry showed a significant negative linear effect decreasing over time. At the same time, we also observed a significant positive quadratic trend, suggesting that concern increased at the end of the testing period. Experiencing fear showed a small decrease over time. For experiencing hope, we did not find any significant linear or quadratic trends.

## Discussion

We sampled over 8.000 Norwegian participants in the first 2 weeks after schools were closed and many employees were sent to work from home, at the beginning of the COVID-19 outbreak in Norway. We observed self-reported health-protective behavior and emotions in real time, while numbers of registered infections rose from 805 to 3399, the number of hospitalized patients rose from 32 to 265, and the number of deceased patients rose from 1 to 14.

Although policy setting may be the main determinant of behavior, psychological factors play an important role in responses to health crises as they modulate how people adopt the guidelines. In the present project we focused on what factors are correlated to engagement in two variants of protective behavior: preventive, such as hygiene behavior, and avoidance, including physical distancing. We employed both a theory- and a data-driven approach, and we explored how attitudes, behavior, and affective reactions changed over the course of the 15-day sampling period. To protect us from overinterpreting spurious effects, which would be costly in the current situation, we set our significance level to *p* < 0.001.

### Information Sources, Confidence in Authorities and Perceived Risk

In our sample, main news sources were news media, government websites, and social media in that order, more than colleagues and family members. When indicating whom they trusted most, participants mainly pointed to the Norwegian Institute of Health (FHI), more so than other Norwegian government sources or European sources. One’s own doctor and hospital was rarely reported as the most trusted source. Confidence was high that authorities, including the Norwegian government, scientists, health professionals, and medical services, were able to manage the outbreak. Despite the increase in infected cases, we observed that confidence stayed stable and even slightly (but not significantly) increased over the time of 15 days.

Respondents expected that they too would likely get infected, with an average above the midpoint on our likelihood rating scale (60%). This average was at the upper end of FHI’s prediction for the general population from 12/03, and higher than the number in Wise et al.’s (2020) sample (*M* = 43) and the representative sample analyzed by Sætrevik (2020), suggesting that due to our sampling strategy our participants might be more concerned and engaged with the topic than the Norwegian population on average. At the same time, perceived severity was predominantly rated with mild or moderate symptoms. Ratings were higher for the perceived likelihood of close others catching the disease (73%) and similarly, a high proportion of respondents (71%) could imagine that someone from their family would show severe symptoms or even die when imagining the worst case. This was also in line with the observations made in parallel by Sætrevik (2020).

### Health-Protective Behavior

Self-reported behavior was very much in line with policies asking for (but not mandating by law) physical distancing and protective hygienic behavior. Even behavior that is sometimes difficult to avoid like taking public transport and going to shops was reported as being reduced or avoided by more than 70% of the sample. More than 70% reported other-protective behavior in the form of reminding other individuals of proper behavior or not visiting older individuals. Fewer people actively helped others by for instance buying groceries or even giving money to charities combating COVID-19. Only a small minority reported irrational avoidant behavior (e.g., avoiding Chinese restaurants – given that the main group bringing infections into Norway were Norwegians coming from winter holidays in the Alps rather than travelers associated with China).

In line with previous findings during other pandemics and also COVID-19 (Bish and Michie, 2010; Harper et al., 2020; Wise et al., 2020), the elevated level of appropriate protective and avoidant behavior was predicted by demographic variables: female participants, higher education levels, and larger household sizes. To some extent, these effects were mediated by elevated perceptions of likelihood and severity of the disease for self and others, but these mediations did not explain much variance and indirect effects were considerably small. These models may underestimate the true effect, however, because expectations and behavior changed over the course of the sampling period. There might be other factors that explain this pattern of results. For instance, recent findings show that compassion and empathy play an important role for the engagement in physical distancing during the COVID-19 outbreak (Pfattheicher et al., 2020) and such reactions have been observed to a higher degree in women (Christov-Moore et al., 2014).

Previous studies also reported that older age predicted more engagement in health-protective behavior. We failed to find a clear replication in the current sample. In fact, our regression analyses point in the direction that older age is associated with less adoption of health-protective behavior. When exploring this association in more detail, we observed four important boundaries. First, we observed a non-linear relationship between age and protective behavior, suggesting that engagement in health-protective behavior increased with age as predicted by previous literature, but then leveled off at around the age of 40–44 and decreased with older age. Second, our sample included only a few participants above the age of 70. Their estimates are therefore highly imprecise compared to younger respondents (that we sampled around 100 times more often) and when excluding age groups with less than 50 participants the relationship between age and protective behavior was reduced to near zero. Third, the effect was reduced when controlling for time. Fourth, when constructing the main outcome variable in a different way in order to account for the possibility that some behaviors from our list were not applicable for older adults (e.g., avoiding work) we observed a weaker effect (see Supplementary Material). Thus, given the composition of our sample we can be more certain that respondents at the age of 40 engage in more protective behavior than respondents at the age of 20. However, whether engagement in health-protective behavior again decreases for individuals at the age of 50 should be interpreted with caution. If this is indeed the case, this would represent an important finding as risk factors and susceptibility increase with age. We recommend testing this question with a representative sample.

In a second step, we tested the influence of more than 30 variables on health-protective behavior employing a supervised machine learning algorithm. We observed that higher engagement in health-protective behavior was associated with (1) larger household size, (2) more children, (3) higher perceived effectiveness of the protective behavior, (4) more media exposure, and (5) reduced belief that ‘too much fuss’ was made about the outbreak (i.e., discrediting the severity and credibility of the crisis, Rubin et al., 2015) above and beyond other factors such as knowledge, perceived risk, living in a municipality with a high amount of recorded cases or one’s own perceived health. The simultaneous presence of demographic and psychological predictors indicates that the psychological mediators of the remaining demographic factors remain unclear.

When considering preventive and avoidance behaviors separately, we observed that household size (i.e., being surrounded by more people) and regarding staying away from others as effective predicted physical distancing, whereas emotional reactions such as concern, worry, or fear and media exposure had a stronger importance in engaging in hygiene behavior. Our findings replicate previous studies suggesting that high perceived effectiveness is important as a predictor of engaging in health-protective, and specifically avoidance behavior (Ajzen and Timko, 1986; Agüero et al., 2011).

The importance of household size and the number of children, especially for the adoption of avoidance behavior, points to the possibility that individuals might feel more personally responsible for their co-habitants. Literature on the effectiveness of health communications suggests that personal relevance represents an important factor for engaging in protective behavior, which is likely higher if more people within one’s social proximity could be affected (Ruiter et al., 2001). Similarly, household size is typically conflated with age showing an inverse u-shaped curve, which fits our observations concerning the association between age and protective behavior. In addition, individuals that need to care for others might show more empathy or compassion, thereby increasing engagement of avoidance behavior as a means of prosociality (Pfattheicher et al., 2020).

On the other hand, engagement in preventive behavior such as hand washing or using hand sanitizing gel was associated less with social-contextual variables, but to a higher degree with felt concern, fear, or worry, as well as increased engagement with the topics. For both types of behaviors, we found that believing there is too much fuss made about the outbreak reduced it. This relation could have several reasons. Wise et al. (2020) identified a subgroup that was disengaged from the news, unaware of risks, and not practicing recommended behavioral change. Participants who indicated that “too much fuss was made” may have belonged to a similar subgroup. On the other hand, there might be a group of people who for some reason cannot change their behavior, and consequently adapt their attitudes to be consistent. In any case, if that group is large enough, it could counteract quarantine measures in communities. It thus seems important to follow up on this effect, again ideally with representative samples.

Contrary to our predictions, we observed that increased confidence in authorities reduced the adoption of health-protective behaviors. Similar findings were observed in the sister study of the current project with an Australian sample (Faasse and Newby, 2020). While confidence in governments, health professionals, and medical services has been reported as crucial for individuals to adopt behavioral change (Bish and Michie, 2010), it is possible that overconfidence results in reckless behavior, as it is assumed that everything will be under control no matter what individual actions are performed. This finding points at a dilemma, as confidence in authorities is needed to establish protective behavior in the first place and reduce panicking or intense fear of the outbreak (Asmundson and Taylor, 2020). Health communications therefore need to highlight the importance of individuals actions as part of greater societal outcomes, and simultaneously communicate conviction in recommended measures and risk.

During the 15-day sampling period, we observed a significant increase in avoidance behaviors. These changes could be explained by individual psychological factors such as increased personal relevance or concern, group behavior and attitudes (such as injunctive norms), or contextual factors. For instance, throughout Norway schools and universities were closed on the 12th of March, creating a uniform behavior change. Similarly, most public events such as sports or concerts were canceled. It is not possible for the present data to show whether changes in avoidance behavior were based on psychological factors or situational constraints. Interestingly, we observed little change in hygiene behavior during the sampling period. It could be possible that hygiene behavior was already quite high at the beginning of data collection: over 90% indicated engaging in more thorough hand washing behavior. On the other hand, increased self-isolation through avoidance behavior could have resulted in neglecting additional preventive behavior.

In contrast to previous studies on responses to pandemics or specifically COVID-19, we failed to find strong associations between perceived risk or knowledge and engagement in protective behavior. While perceived likelihood and severity showed positive relations with health-protective behavior, these effects were considerably small and smaller than factors such as the number of children or experienced concern. Similarly, knowledge showed no or even a negative relationship with engagement in protective behavior. As knowledge and media exposure were on average quite high, it could be that we simply did not have enough variation in the sample to detect a larger effect. Nevertheless, the implication seems to be that motivating people to practice protective behavior works best by emphasizing that it is effective, rather than by exaggerating risks of not engaging in it.

The present findings mostly replicate an earlier study using nearly identical methods in an Australian sample in an earlier stage of the pandemic (Faasse and Newby, 2020). Similar to this study, we found positive relations to media exposure, concern and worry, as well as effectiveness of behavior. In addition, we also replicated the finding that confidence in authorities and believing that too much fuss was made resulted in less health-protective behavior.

Our observed effect sizes ranged from zero-order correlations (*r*) of 0.26 between concern/worry and health-protective behavior to standardized regression coefficients (beta) of 0.07 for the prediction by media exposure when controlling for the other variables, or less. The estimated effect sizes are in line with published literature focusing on attitude-behavior relationships (Bosco et al., 2015) and can be considered as small to medium effects. Similarly, our effects are comparable to previous research exploring predictors of health-protective behavior during the COVID-19 outbreak (Faasse and Newby, 2020; Harper et al., 2020; Wise et al., 2020). It would have been helpful to define a smallest effect size of interest in order to be able to conclude when an effect is absent by for example applying equivalence testing (Lakens, 2017). However, given the exponential nature of the growth of infections it is difficult to decide on a cut-off regarding which effects might not be of practical importance anymore. While standardized regression or correlation coefficients of 0.05 might be typically considered as too small to be of practical importance, they could still be informative in the current context. Answers to that can only come from models that integrate behavior and epidemiological effects (e.g., Poletti et al., 2012). In general, we note that our effects were on average comparable small.

### Limitations

Our study has several limitations that should be considered when interpreting the findings. First, although large, our sample was not collected in a way that makes it representative. Women, younger people, and individuals with a higher education level are overrepresented; this should be taken into account when interpreting the presented findings. Nevertheless, our total sample size was large enough that we trust our estimates for male participants. Notably, percentage of people expecting to become infected, confidence in the government to handle the crisis, and percentage of those worried about family members are similar to numbers found in two representative survey studies among the Norwegian population (Kantar, 2020a,b), suggesting that our sample might be quite similar to the Norwegian population at large. Nevertheless, our study provides a snapshot of a 15-day period, focusing on a non-representative sample representing a specific culture with all its societal and normative implications, as well as certain healthcare systems and authorities that are hardly generalizable to different countries, healthcare systems, or timepoints in a pandemic.

Second, although time is a meaningful variable in the 15 days window that we observed, our sample is cross-sectional, not longitudinal. Changes over time can thus be caused by various confounding variables and simply be due to sampling variation, despite our efforts to control for that. Strong inferences about intra-individual change need repeated measures in a longitudinal design, which we do not have (Borsboom et al., 2003; Fisher et al., 2018).

Third, we did not pre-register our research methods and analysis plan. Indeed, we largely adopted an existing instrument and developed the literature review and hypothesis in parallel to data collection. The main research scope of the present project was exploratory in nature and we did our best to increase the reliability of our findings by conducting a split-half validation method (IJzerman et al., 2018). Due to the exploratory approach, we included several variables that have been found to predict protective behavior in past literature or were deemed important. Of course, it is possible that we failed to include important variables associated with health-protective outcomes, such as compassion or empathy (Pfattheicher et al., 2020).

Fourth, the measurement of some of the included variables, especially our outcome variable, could be improved. In the current project we assessed protective behavior using a dichotomous format (answer alternatives yes/no, we also added unsure, and not applicable). A Likert-scale type measurement might be superior in capturing the whole breadth of responses in the outcome variable. At the moment a respondent will answer yes if she avoided specific situations once or several times within the last 2 weeks. Using more response options would allow us to differentiate among such responses. Similarly, we focused on self-report of behavior, not actual behavior and there might be a gap between reported and actual health-protective behavior. However, recent research focusing on GPS movement data in the US during the COVID-19 outbreak suggests that self-report data might be used as a proxy for actual behavior (Gollwitzer et al., 2020).

Our measures of protective and avoidant behavior were much more comprehensive than our measure of other-supporting behavior. As the crisis proceeds, various behaviors that support the community through donating food, equipment, and money, making masks, supporting each other through buying food, and taking care of children become important, and it is known that such communal behavior emerges in crises and can be stifled by authorities reacting the wrong way (Solnit, 2009; Drury et al., 2019). Future studies should place more emphasis on such measures.

Finally, we believe that our understanding of the motives behind protective and avoidant behavior is not ideal. Unless one knows for sure whether oneself or another person is infected, most behavior serves both to protect oneself and others. For instance, the discussion about wearing non-clinical facial masks has moved from initial arguments that they are not providing total protection for the wearer to the insight that they do protect others if the wearer is infected - and if everybody protects everybody else, then everybody is protected. In our data, we are not able to tease apart motivation to protect the self and other-protection motivation, either for close others or the community. Again, this remains a crucial topic for future work.

## Conclusion and Outlook

The present project provides a snapshot of individuals’ attitudes, behavioral actions, and affective reactions during 2 weeks following the COVID-19 outbreak in Norway. While our findings do not generalize to the whole Norwegian population, nor to other countries with different courses of action responding to the outbreak or different healthcare systems, they provide important information on the nature of what psychological and demographic variables might influence health-protective behavior and how such variables change over time. The findings can provide insights and indications in order to improve healthcare communications:

(1) Perceptions of effectiveness of protective behavior are important; they emerge as crucial especially when trying to predict physical distancing. They could be increased

by tailoring communication strategies to various groups, emphasizing how different people can engage in effective preventive (hygienic) or avoidance (distancing) behavior.
(2) People differ, and these differences matter for the adoption of protective behavior: being female, household size, and number of children all seem to play a role. On one hand, these factors point to how early on in a crisis first changes can be reached quickly by targeting such response groups. On the other hand, this again shows that tailored messaging and targeted behavior change campaigns are indicated.
(3) Physical distancing and hygiene seem to be driven by somewhat different factors: the former more by social variables and beliefs of effectiveness, the second more by emotional processes. Again, campaigns targeting these complementary protections should be aware of that.
(4) In line with previous literature, there is a subset of the population that discredits severity and credibility of the crisis, indexed in our study as the belief that “too much fuss is being made” about this, which is in turn associated with less engagement in health-protective actions (cf. Rubin et al., 2015). It may be fruitful to model and investigate the potential impact such individuals can have on the spread of the disease, the reasons for their beliefs, and targeted ways to change their beliefs.

Finally, the present project highlights that although similar factors can be found across different countries or medical systems that seem to influence protective outcomes (e.g., Harper et al., 2020; Wise et al., 2020), it is important to take the specific trajectories and developments in each country or healthcare systems into account to be able to successfully model and identify important variables predicting health-protective behavior (see Mækelæ et al., 2020).

## Data Availability Statement

The datasets presented in this study can be found in online repositories. The names of the repository/repositories and accession number(s) can be found in the article/ Supplementary Material.

## Ethics Statement

The studies involving human participants were reviewed and approved by Internal Review Board, Institute of Psychology, University of Oslo. The patients/participants provided their written informed consent to participate in this study.

## Author Contributions

JG and KF devised the original method. JZ, TS, and AH adapted the original instrument for Norway, and AH translated it into Norwegian. JZ analyzed the data and wrote the first draft. All authors contributed to revisions.

## Conflict of Interest

The authors declare that the research was conducted in the absence of any commercial or financial relationships that could be construed as a potential conflict of interest.

## Footnotes

1 Similar to previous studies (e.g., Pfattheicher et al., 2020), we employ the term *physical distancing* instead of *social distancing* to highlight the fact that individuals are advised to keep a *physical* distance.

2 https://osf.io/crs2n/

3 https://www.vg.no/spesial/2020/corona/

4 In a first version of the questionnaire the scale went from “strongly agree (1)” to “strongly agree (5)” due to a clerical error. This was corrected immediately after the first approximately 20 responses.

5 At the time the survey was started, symptoms like nausea or diarrhea were not regarded as typical symptoms of COVID-19. However, as the outbreak proceeded the WHO added these to the list of possible symptoms (https://www.who.int/health-topics/coronavirus#tab=tab_3). Thus, these responses were not part of the final knowledge score.

6 During the time the study was conducted there was a general recommendation that face masks should only be worn by infected people. This recommendation changed after the project was terminated, highlighting the usefulness of face masks also for healthy people.

7 https://osf.io/utk5y/

8 Originally, we subsumed these under the label of *counterproductive behavior*. However, as the outbreak progressed and more people got infected, wearing a face mask if infected could be considered a behavior protective of others’ health. Similarly, we added *prosocial* as an own category, but most physical distancing behavior can be regarded as prosocial in the long run (e.g., Pfattheicher et al., 2020).
