# Supplementary material for: Correlates of Health-Protective Behavior During the Initial Days of the COVID-19 Outbreak in Norway
Source: Front Psychol. 2020 Oct 6;11:564083. doi: 10.3389/fpsyg.2020.564083 (PMC7573186; doi:10.3389/fpsyg.2020.564083)
Supplement: Supplementary file 1 [file Data_Sheet_1.PDF]

Correlates of Health-Protective Behavior During the Initial Days of the COVID-19 Outbreak in Norway

Supplementary Material

Janis H. Zickfeld[1,2], Thomas W. Schubert[1], Anders Herting[1], Jon Grahe[3] & Kate Faasse[4]

[1]University of Oslo, [2]University of Mannheim, [3]Pacific Lutheran University, [4]University of New South Wales

Table S1. Overview of items and mean/sum-scores.

S1.1 Continuous variables

Item code	Item Label	Min	Max	Mean	SD	Median
Media Exposure	Mean score Q8-Q10	1	6	4.80	.75	5
Q8	How much information have you seen, read, or heard about COVID-19?	1	4	3.709443	0.488781	4
Q9	How much do you think you know about COVID-19?	1	4	2.941197	0.641436	3
Q10	How closely have you been following news about the recent outbreak of COVID-19?	0	10	7.759691	1.61246	8

Confidence in Authorities	Mean score Q14, Q16, Q101, Q22	0	10	6.07	1.64	6.25
Q14	How confident are you that the Norwegian government is providing full and accurate information about COVID-19?	0	10	6.929082	2.130756	7
Q16	To what extent do you believe that scientists and other medical and health experts understand COVID-19?	0	10	6.569468	1.780454	7
Q101	How confident are you that health authorities will be able to manage COVID-19?	0	100	54.77505	23.6345	58
Q22	How confident are you that hospitals and medical services can provide adequate treatment for people infected	0	100	52.91912	24.5819	54

	with the virus?					
Concern/Worry (Q17)	How concerned or worried are you about the COVID-19 outbreak?	1	5	3.094105	0.909299	3
Perceived Risk Likelihood (Q18a)	How likely do you think it is that you, personally, will catch COVID-19?	0	100	60.34051	22.26784	60
Perceived Risk Close Others Likelihood (Q18b)	How likely do you think it is that somebody from your family or a close friend will catch COVID-19?	0	100	72.84894	22.53088	78
Perceived Risk Severity (Q21a)	If you do catch COVID-19, how serious do you think your symptoms will be?	1	6	2.804477	0.818201	3
Perceived Risk Close Others Severity (Q21b)	If you think that a family member or close friend might get infected. what do you think the worst possible	1	6	4.594095	1.319176	5

	outcome for that person could be?					
Effectiveness of Behavior (Q20_1)	How much can you, personally, do to protect yourself from catching the virus?	0	1	77.42876	16.97985	80
Fuss (Q23)	To what extent do you agree with the following statement “too much fuss is being made about the risk of COVID-19”	1	5	1.836884	1.117636	1
Confidence in the Chinese government (Q15)	How confident are you that the Chinese Government is providing full and accurate (based on what they currently know) information about the COVID-19 coronavirus outbreak?	0	10	4.341947	2.264053	4
Q100_1	Was there already a moment when you wondered whether	0	100	43.59477	35.9732	40

	you yourself were infected?					
Fear	Mean score Q23a_1, Q23a_2, Q23a_3	1	5	3.61	1.00	4
Q23a_1	Thinking of COVID-19, I feel fearful	1	5	3.429066	1.191712	4
Q23a_2	Thinking of COVID-19. I feel frightened	1	5	3.631328	1.146235	4
Q23a_3	Thinking of COVID-19, I feel anxious	1	5	3.772082	1.08882	4
Hope	Mean score Q23a_4, Q23a_5, Q23a_6	1	5	2.46	.88	2.33
Q23a_4	Thinking of COVID-19, I feel optimistic	1	5	2.719454	1.171676	3
Q23a_5	Thinking of COVID-19, I feel encouraged	1	5	1.682597	0.966675	1
Q23a_6	Thinking of COVID-19, I feel hopeful	1	5	2.9939	1.213495	3
Relaxation (Q23a_7)	Thinking of COVID-19, I feel relaxed	1	5	2.140975	1.124874	2

Anger	Mean score of Q23a_8, Q23a_9	1	5	2.26	1.16	2
Q23a_8	Thinking of COVID-19, I feel furious	1	5	2.478985	1.321135	2
Q23a_9	Thinking of COVID-19, I feel outraged	1	5	2.055376	1.211233	2
Sadness	Mean score of Q23a_10, Q23a_11	1	5	3.11	1.05	3
Q23a_10	Thinking of COVID-19, I feel depressed	1	5	2.63594	1.311309	3
Q23a_11	Thinking of COVID-19, I feel sad	1	5	3.580287	1.165509	4

S1.2 Media sources (checkboxes)

Item Code	Item Label	N (checked)	% (checked)
Information sources	How have you been getting information about the COVID-19 coronavirus outbreak? (Check all that apply)		
Q12_1	News media (e.g. newspapers, online news websites, television news coverage)	8688	91.1
Q12_2	Social media (e.g. twitter, facebook)	5793	60.7
Q12_3	Official government websites (e.g. Centres for Disease Control	7516	78.8

	and Prevention, World Health Organisation)		
Q12_4	Family member(s)	2860	30.0
Q12_5	Colleague(s) or friend(s)	3799	39.8
Q12_6	None of the above	10	0.1
Q12_7	Other sources of information (please enter):	940	9.9
Trust in sources	Which one of these sources do you trust most to advise you about the COVID-19 coronavirus outbreak in Norway?		
Q13_1	My doctor / GP	916	9.6
Q13_4	European Centre for Disease Control and Prevention (ECDC)	3045	31.9
Q13_5	My local hospital	1107	11.6
Q13_6	The Norwegian Government	3085	32.3
Q13_7	None of the above	275	2.9
Q13_8	Other	728	7.6
Q13_9	My State Department of Health	3424	35.9
Q13_10	Folkehelseinstitut (FHI)	8021	84.1
Q13_11	(Norwegian) Media	1782	18.7

S1.3 Knowledge

Item Code	Item Label	Correct (1)		Wrong or unsure (0)	
		n	%	n	%
Knowledge	Sum score Q24_1:Q28				

Q24_1	There is a vaccine to protect against COVID-19 coronavirus (1)	7586	91.08 %	743	8.92 %
Q24_2	There is an effective medicine available for treating COVID-19 coronavirus (2)	7031	84.46 %	1294	15.54 %
Q24_3	It it is likely that some people will have natural immunity to COVID-19 coronavirus (4)	2685	32.26 %	5638	67.74 %
Q24_4	The seasonal flu vaccine will protect me from COVID-19 coronavirus (5)	7831	94.01 %	499	5.99 %
Q24_5	The health effects of COVID-19 coronavirus appear to be more severe for people who already have a serious medical condition (9)	8202	98.55 %	121	1.45 %
Q24_6	The health effects of COVID-19 coronavirus appear to be more severe in children	6796	81.63 %	1529	18.37 %

	and pregnant women (23)				
Q24_7	Domestic pets can be infected with and spread COVID-19 coronavirus (10)	3040	36.50 %	5288	63.50 %
Q24_8	Antibiotics are an effective treatment for COVID-19 coronavirus (11)	7251	87.04 %	1080	12.96 %
Q24_9	Packages or letters from China can spread the virus (12)	5409	64.93 %	2922	35.07 %
Q24_10	Taking vitamin C or other vitamins will protect me from the COVID-19 coronavirus (13)	6784	81.42 %	1548	18.58 %
Q24_11	Spraying chlorine on my body will protect me even if the COVID-19 coronavirus has already entered my system (14)	8120	97.46 %	212	2.54 %
Q24_12	Vaccines against pneumonia will protect me against the COVID-19 coronavirus (15)	6287	75.52 %	2038	24.48 %

Q24_13	Regularly rinsing my nose with saline will protect me against the COVID-19 coronavirus (16)	7346	88.20 %	983	11.80 %
Q24_14	The virus was genetically engineered as part of a biological weapons program (20)	7096	85.22 %	1231	14.78 %
Q24_15	The virus was deliberately released rather than naturally occurring (21)	6934	83.26 %	1394	16.74 %
Q24_16	The number of people who have really been infected with COVID-19 coronavirus is much higher than has been officially reported (22)	735	8.83 %	7587	91.17 %
	To your knowledge, what are the most common symptoms of the COVID-19 coronavirus?				
Q25_1	Fever (1)	8176	98.64 %	113	1.36 %
Q25_2	Cough (2)	8118	97.91 %	173	2.09 %
Q25_3	Sore throat (3)	497	5.99 %	7795	94.01 %

Q25_4	Shortness of breath (4)	8046	97.02 %	247	2.98 %
Q25_5	Nausea (5)	5698	68.69 %	2597	31.31 %
Q25_6	Vomiting (6)	6835	82.59 %	1441	17.41 %
Q25_7	Diarrhea (7)	4419	53.36 %	3862	46.64 %
Q27	To minimize the transmission of COVID-19. who should be wearing a face mask?	6013	72.73 %	2254	27.27 %
Q28	To your knowledge approximately what percentage of people who have been infected have died from the virus?	5287	63.88 %	2990	36.12 %

S1.4 Health protective behaviors

		Engaged in (1)		Not engaged in, unsure, or not applicable (0)	
		n	%	N	%
Health Protective Behavior	Sum score Q33_1:Q33_10, Q33_12:Q33_17, Q33_19:Q33_24				
Physical Distancing	Sum score Q33_1:Q33_10; Q33_19:Q33_21				

Hygiene Behavior	Sum score Q33_12:Q33_17				
Q33_1	Reduced or avoided going to work or university (1)	5952	82.79 %	1237	17.21 %
Q33_2	Reduced or avoided using public transport (2)	6445	90.67 %	663	9.33 %
Q33_3	Reduced or avoided flying domestically (3)	4484	91.27 %	429	8.73 %
Q33_4	Reduced or avoided flying internationally (4)	4663	91.88 %	412	8.12 %
Q33_5	Reduced or avoided going to public events such as movies. sporting events, or concerts (5)	7291	97.17 %	212	2.83 %
Q33_6	Self-isolated at home (17)	6048	77.01 %	1806	22.99 %
Q33_7	Reduced or avoided going to hospitals or going to the doctor unless absolutely necessary (6)	4571	89.24 %	551	10.76 %
Q33_8	Reduced or avoided going into shops (7)	6828	84.28 %	1274	15.72 %
Q33_9	Reduced or avoided staying in hotels, hostels, or Airbnbs (8)	3992	88.69 %	509	11.31 %

Q33_10	Reduced or avoided sending your children to school or childcare (9)	3008	90.33 %	322	9.67 %
Q33_11	Reduced or avoided going to Chinese restaurants, Chinatowns, or Chinese precincts (10)	744	15.37 %	4096	84.63 %
Q33_12	Cleaned or disinfected things you might touch (such as doorknobs or hard surfaces) more often than usual (11)	5690	70.60 %	2370	29.40 %
Q33_13	Used sanitizing hand gel to clean your hands more often than usual (12)	6541	80.95 %	1539	19.05 %
Q33_14	Washed your hands more thoroughly than usual (13)	7955	97.36 %	216	2.64 %
Q33_15	Washed your hands more often than usual (18)	8005	97.90 %	172	2.10 %
Q33_16	Reduced or avoided touching your face (19)	3471	48.89 %	3629	51.11 %
Q33_17	Tried to cough or sneeze into the crook of your arm or	7473	97.60 %	184	2.40 %

	inner elbow (20)				
Q33_18	Worn a face mask when going out in public (14)	166	2.05 %	7924	97.95 %
Q33_19	Prepared for possible quarantine e.g. by purchasing extra supplies (16)	2998	37.48 %	5002	62.53 %
Q33_20	Tried to stay at least 3 feet away from anyone who is coughing or sneezing (21)	7767	98.04 %	155	1.96 %
Q33_21	Avoided visiting elderly people or people with preexisting health problems to protect them from infection (22)	6696	96.33 %	255	3.67 %
Q33_22	Helped buying groceries and supplies for people who are in quarantine (23)	1570	21.52 %	5724	78.48 %
Q33_23	Talked to others to remind them of behavior that avoids infection (24)	6776	85.37 %	1161	14.63 %
Q33_24	Given money to the Red Cross or other charities to help them	779	9.90 %	7089	90.10 %

fight the disease (25)

S1.5 Nominal variables

Item Code	Item Label	Value	N	%
Municipality (Q86)	Which municipality do you live in?	Oslo'	3528	38.1
		Rogaland'	466	5.0
		Møre og Romsdal'	258	2.8
		Nordland'	199	2.2
		Viken'	1866	20.2
		Innlandet'	467	5.0
		Vestfold og Telemark'	526	5.7
		Agder'	349	3.8
		Vestland'	738	8.0
		Trøndelag'	607	6.6
		Troms og Finnmark'	250	2.7
Age (Q40)	Which of the following age groups do you belong to?	18-19'	106	1.3
		20-24'	827	9.9
		25-29'	1382	16.6
		30-34'	1255	15.1
		35-39'	1100	13.2
		40-44'	1020	12.3
		45-49'	918	11.0
		50-54'	704	8.5
		55-59'	448	5.4
		60-64'	270	3.2
		65-69'	173	2.1
		70-74'	84	1.0
		75-79'	19	0.2

		80+'	8	0.1
Gender (Q41)	What is your gender?	Male'	1854	22.0
		Female'	6463	76.9
		Non-binary'	40	0.5
		Different identity (please state):'	20	0.2
		Prefer not to say'	32	0.4
Children (Q42)	How many children do you have?	None	3933	46.8
		1	1277	15.2
		2	2084	24.8
		More than 2	1114	13.2
Education level (Q74)	What is the highest level of education you have completed?	Less than high school'	192	2.3
		High school graduate'	1301	15.5
		Some college'	1392	16.6
		2 year degree'	2277	27.1
		4 year degree'	2136	25.4
		Professional degree'	765	9.1
		Doctorate'	344	4.1
Community Type (Q84)	What type of community do you live in?	Large city'	4417	52.5
		Rural area'	955	11.4
		Suburb near a large city'	1150	13.7
		Small city or town'	1886	22.4
Household Size (Q96)	Including yourself, how many people live	1 (just me)'	1596	19.0
		2'	2843	33.8
		3'	1603	19.1

	in your household?	4'	1604	19.1
		5 or more people'	761	9.1
Health (Q43)	In general, would you say your health is:	Poor'	374	4.4
		Fair'	952	11.3
		Good'	3107	37.0
		Very good'	2766	32.9
		Excellent'	1208	14.4
Vaccine (Q39)	Have you had a flu vaccine in the past year?'	Yes'	2280	27.1
		No'	6103	72.6
		Unsure'	25	0.3
Area (Q86)	Within the past two weeks, have you been in an affected area where there is high transmission of COVID-19	Yes'	1574	18.8
		No'	5591	66.7
		Unsure'	1222	14.6
Contact (Q44)	Within the past two weeks, have you been in close contact with one or more people who are known or suspected to be infected with COVID-19?	Yes'	1157	13.8
		No'	5707	68.0
		Unsure'	1523	18.2
Symptoms (Q93)	Within the past two weeks, have you experienced any symptoms of COVID-19?	Yes'	2371	28.3
		No'	4490	53.5
		Unsure'	1525	18.2
Symptoms Other (Q94)	Within the past two weeks, have your close friends	Yes'	3127	37.3
		No'	4015	47.9

	or family experienced any symptoms of COVID-19?	Unsure'	1243	14.8
Chronic illnesses and/or health problems (Q85)	Do you have one or more chronic illness or health problem that increases your risk of infection or complications from COVID-19 coronavirus?	Yes'	1581	18.9
		Prefer not to say'	41	0.5
		Unsure'	820	9.8
		No'	5945	70.9
Chronic illnesses and/or health problems Other (Q85)	Do any of your close friends or family members have one or more chronic illnesses or health problems that increases their risk of infection or complications from COVID-19 coronavirus?	Yes'	6338	75.6
		No'	1415	16.9
		Unsure'	602	7.2
		Prefer not to say'	32	0.4

Mediation Models

For the mediation models, we employed a bootstrapping method (*n*=1000) to calculate confidence intervals around the indirect effect. We estimated four different mediation models using health-protective behavior as the outcome and perceived risk likelihood and severity as the mediators with gender, age, or education level as the predictor. In addition, we employed perceived risk likelihood and severity of close others and household size as a mediator in a fourth model. Although we did not pre-register these models, there were derived from the literature cited above. For gender, we found positive but statistically non-significant indirect effects for both likelihood and severity. Focusing on age, we observed a negative significant

indirect effect through likelihood, and a positive significant indirect effect through severity. For education level, we observed a significant positive indirect effect for likelihood and a significant negative indirect effect for severity. Finally, for household size we found a significant positive indirect effect through likelihood of close others but only a very small indirect effect for severity of close others, failing to reach statistical significance. For all mediation models, we observed partial mediations. All four demographic variables still predicted health-protective behavior to a considerable degree when including the mediators. An overview of results is provided in Figure S1.

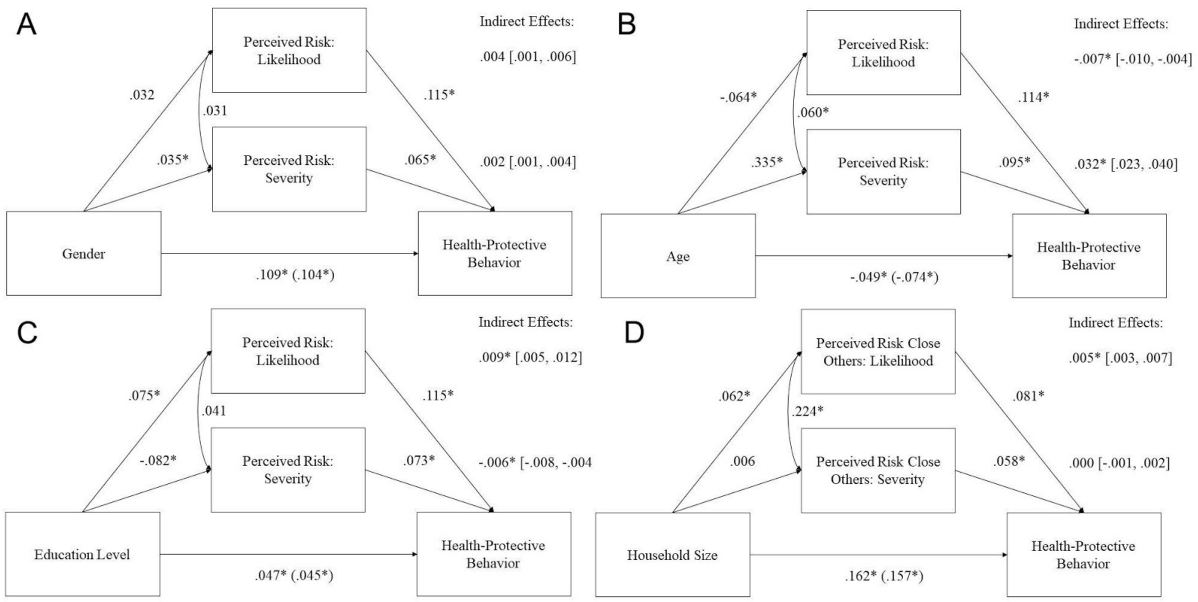

Figure S1. Mediation diagrams using gender (A; 0 = male, 1 = female), age (B), education level (C), and household size (D) as predictors, perceived risk likelihood and severity (A,B,C) or perceived risk likelihood and severity close others (D) as mediators, and health-protective behavior as the outcome. Estimates represent standardized estimates, confidence intervals are 95% bootstrap CIs. *indicates <.001.

Variables predicting health-protective behavior

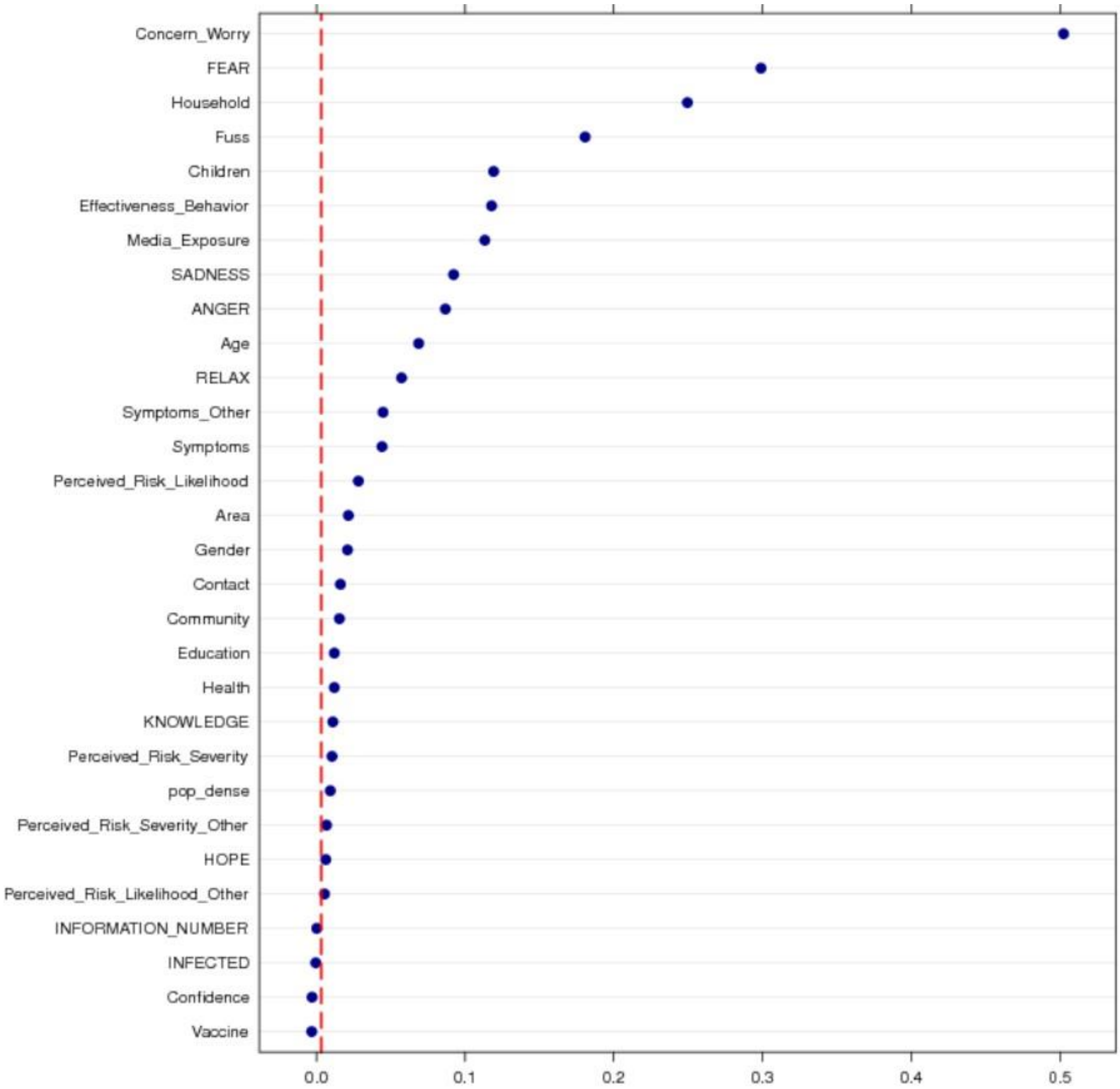

Figure S2. Permutation variable importance of predictors of health-protective behavior (seed 1, MTry = 5, ntree = 1000). Variables exceeding the red line are unlikely to result from random noise.

Variables predicting physical distancing

Replications were stable with Spearman rank correlations between .95 and .97.

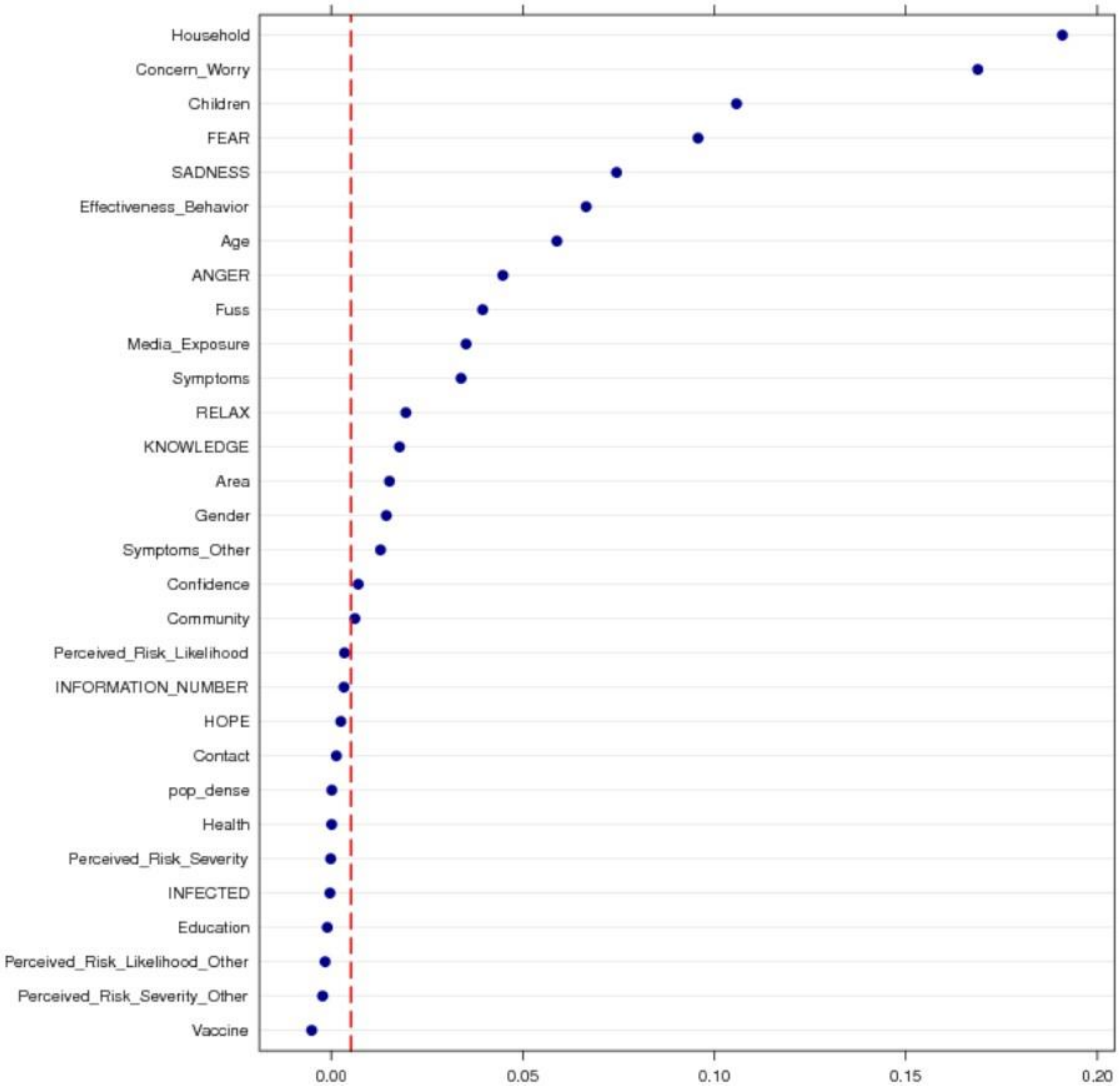

Figure S3. Permutation variable importance on physical distancing (outcome; MTry = 5, seed = 1, ntree = 1000).

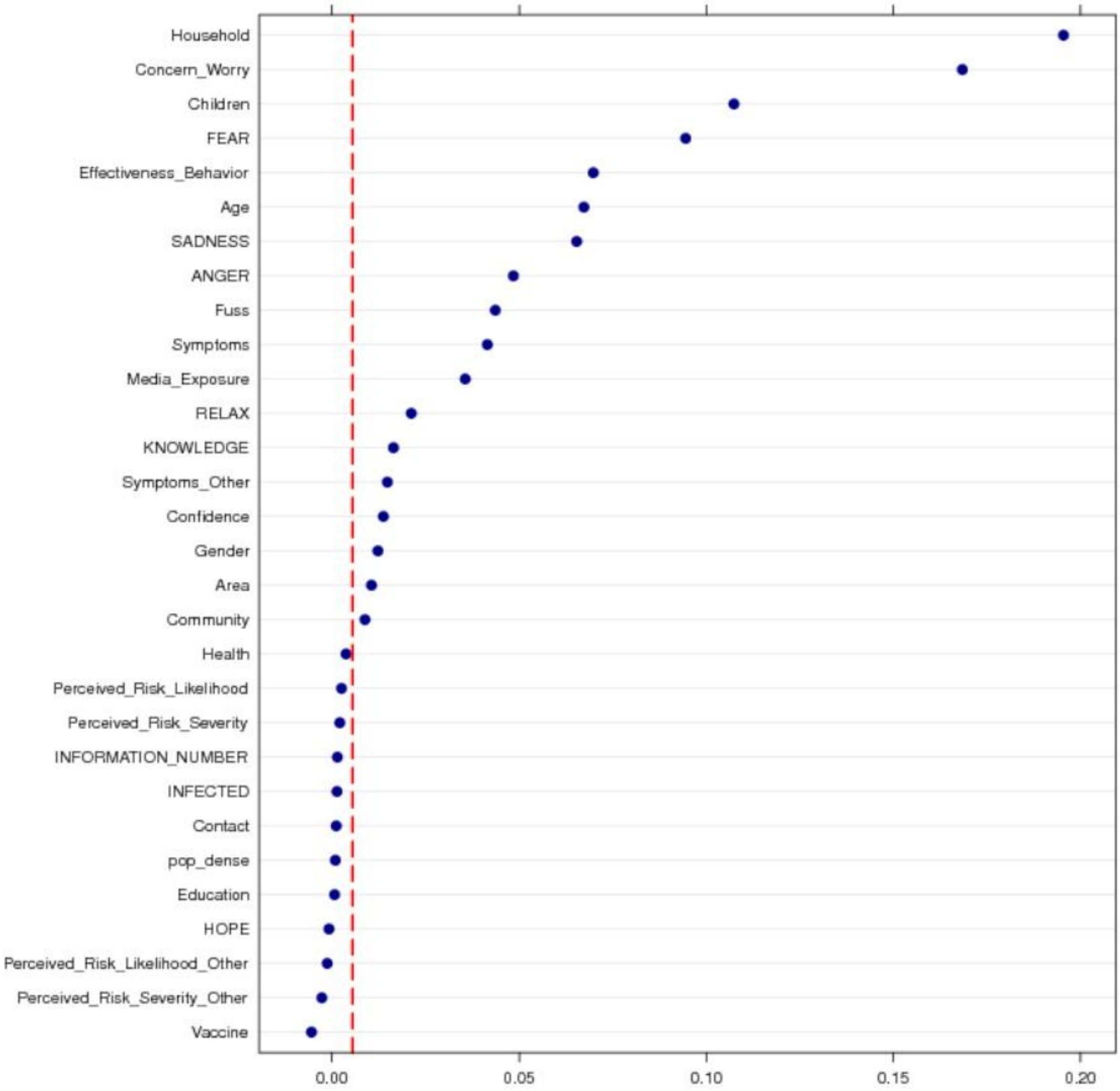

Figure S4. Permutation variable importance on physical distancing (outcome; MTry = 5, seed = 2, ntree = 1000).

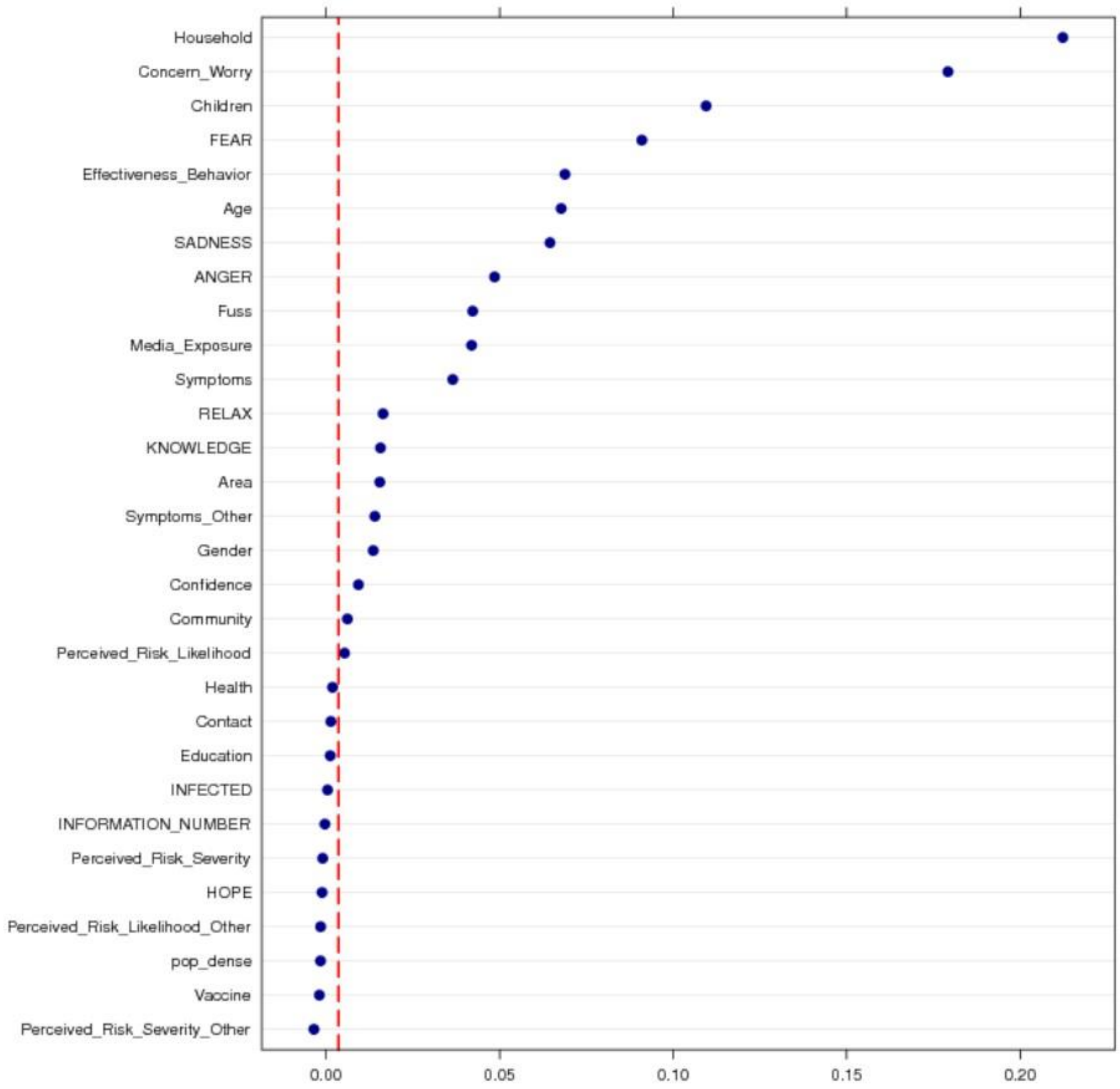

Figure S5. Permutation variable importance on physical distancing (outcome; MTry = 6, seed = 1, ntree = 1000).

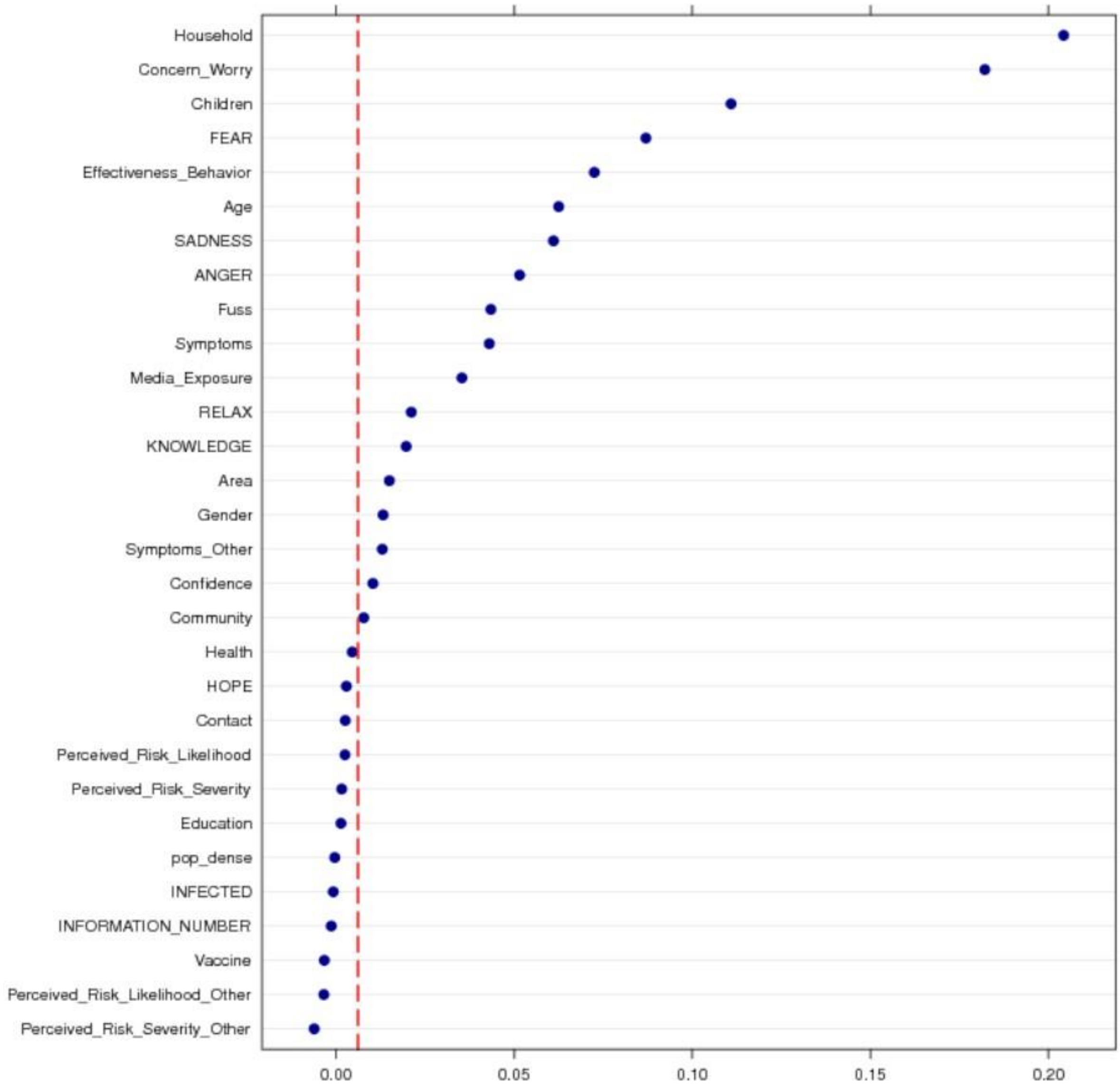

Figure S6. Permutation variable importance on physical distancing (outcome; MTry = 6, seed = 2, ntree = 1000).

Table S2 *Regression results using SOCIAL_DISTANCING as the criterion.*

Predictor	*b*	*b* 95% CI [LL, UL]	*beta*	*beta* 95% CI [LL, UL]	*r*	Fit
(Intercept)	5.90*	[4.55, 7.26]				
Household Size	0.26*	[0.16, 0.37]	0.13	[0.08, 0.18]	.21	
Concern/Worry	0.16	[0.02, 0.31]	0.06	[0.01, 0.11]	.18	
Children	0.32*	[0.18, 0.46]	0.14	[0.08, 0.20]	.13	
Fear	0.13	[-0.01, 0.27]	0.05	[-0.00, 0.10]	.17	
Sadness	0.01	[-0.10, 0.13]	0.00	[-0.04, 0.05]	.10	
Perceived	0.01*	[0.01, 0.02]	0.09	[0.05, 0.13]	.08	

Effectiveness						
Age	-0.14*	[-0.19, -0.08]	-0.13	[-0.18, -0.08]	-.10	
Anger	0.06	[-0.03, 0.16]	0.03	[-0.01, 0.07]	.10	
Fuss	-0.18*	[-0.28, -0.08]	-0.07	[-0.11, -0.03]	-.14	
Media Exposure	0.16	[0.02, 0.30]	0.04	[0.01, 0.08]	.10	
Symptoms	0.48*	[0.25, 0.71]	0.09	[0.04, 0.13]	.14	
Relaxation	-0.08	[-0.18, 0.03]	-0.03	[-0.08, 0.01]	-.14	
Knowledge	-0.04	[-0.08, -0.00]	-0.04	[-0.08, -0.01]	-.03	
Area	0.19	[-0.05, 0.43]	0.03	[-0.01, 0.07]	.03	
Gender	0.26	[0.02, 0.49]	0.04	[0.00, 0.08]	.09	
Symptom Close Other	0.09	[-0.13, 0.31]	0.02	[-0.02, 0.06]	.10	
Confidence in Authorities	-0.04	[-0.11, 0.02]	-0.03	[-0.06, 0.01]	-.06	
						R^2 = .126*<br>95% CI[.10,.14]

Note. A significant *b*-weight indicates the beta-weights are also significant. *b* represents unstandardized regression weights. *beta* indicates the standardized regression weights. *r* represents the zero-order correlation. *LL* and *UL* indicate the lower and upper limits of a confidence interval, respectively.
* indicates $p < .001$.

Variables predicting hygiene behavior

Replications were stable with Spearman rank correlations between .95 and .97.

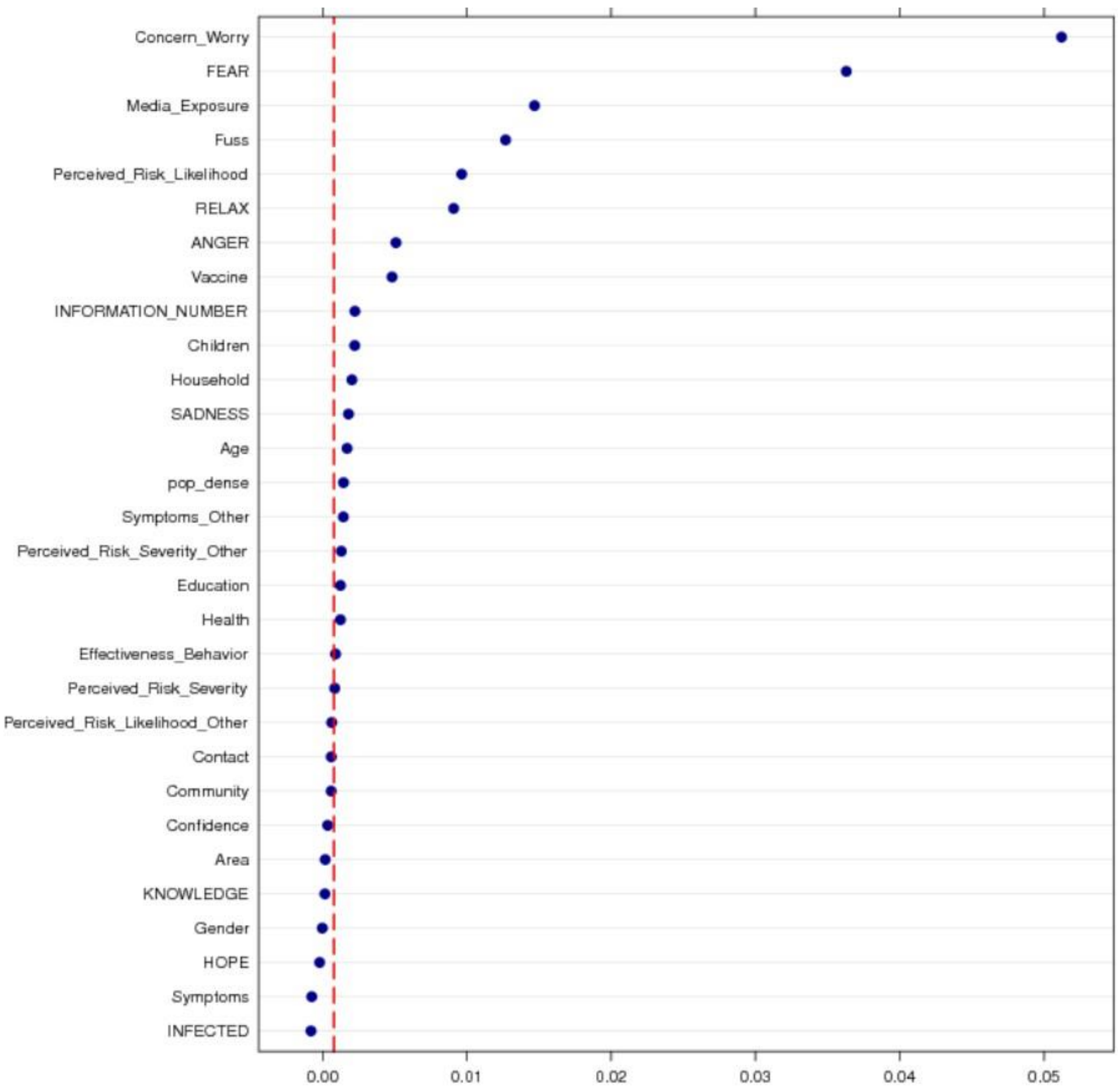

Figure S7. Permutation variable importance on hygiene behavior (outcome; MTry = 5, seed = 1, ntree = 1000).

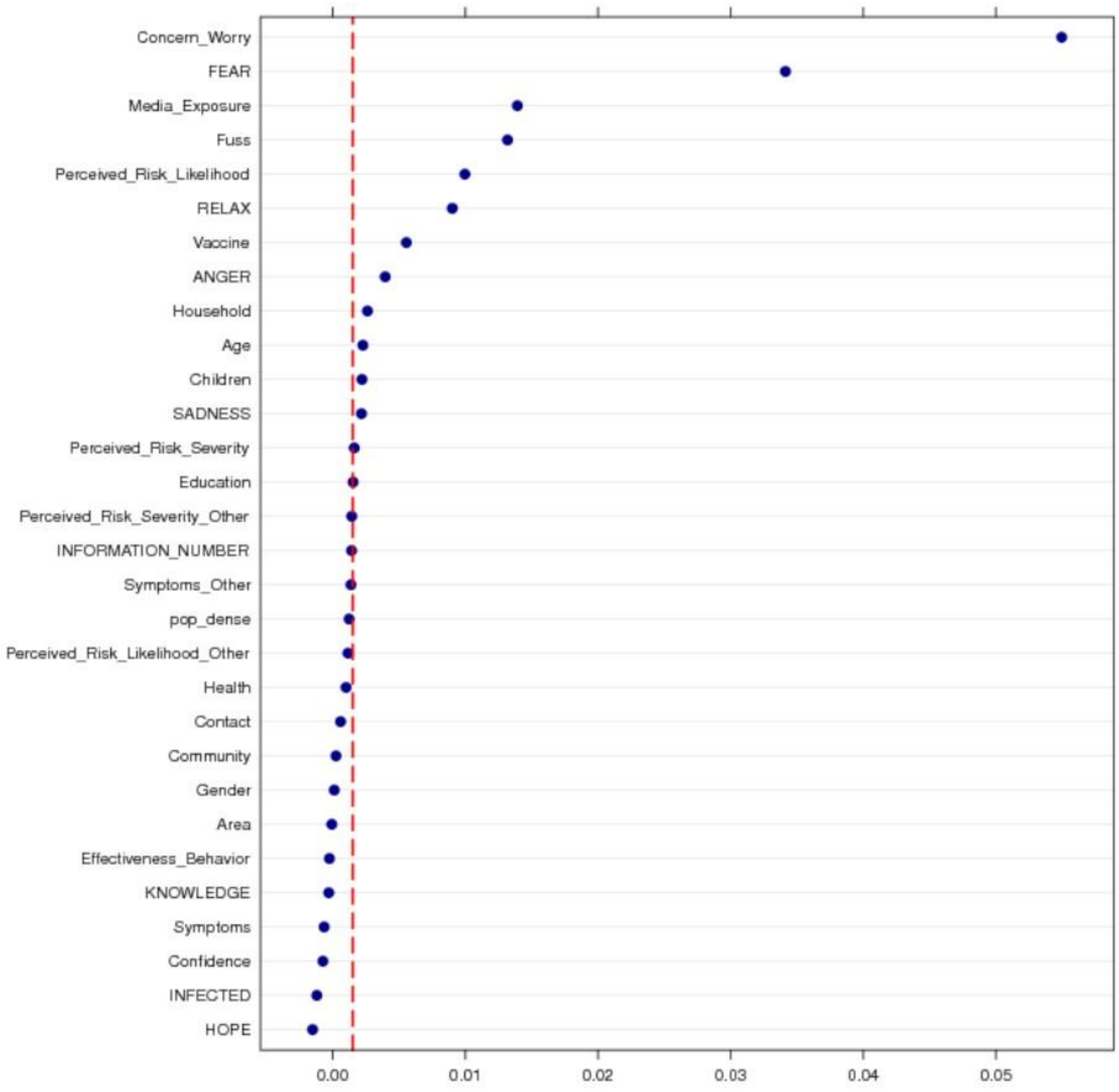

Figure S8. Permutation variable importance on hygiene behaviour (outcome; MTry = 5, seed = 2, ntree = 1000).

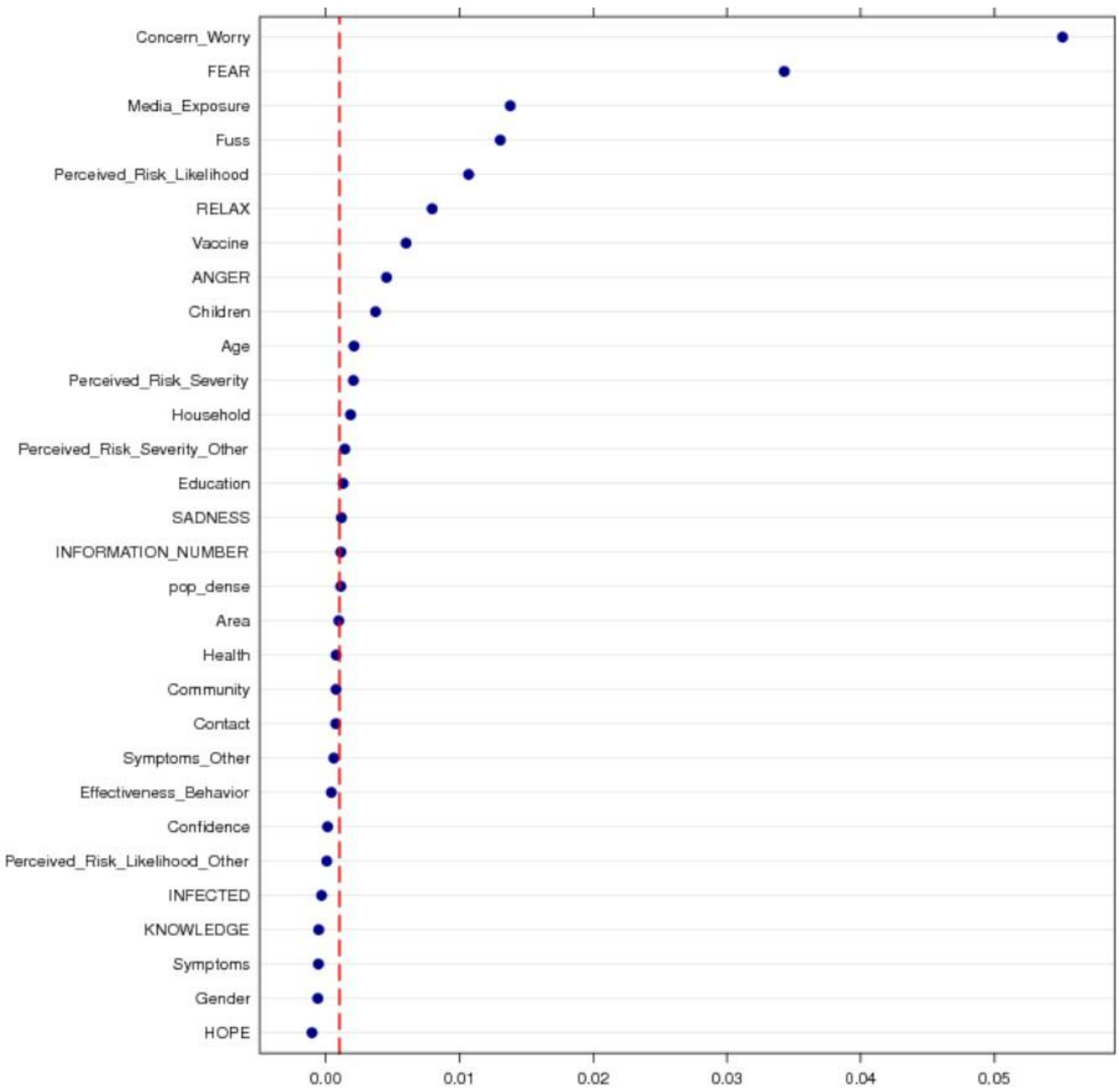

Figure S9. Permutation variable importance on hygiene behaviour (outcome; MTry = 6, seed = 1, ntree = 1000).

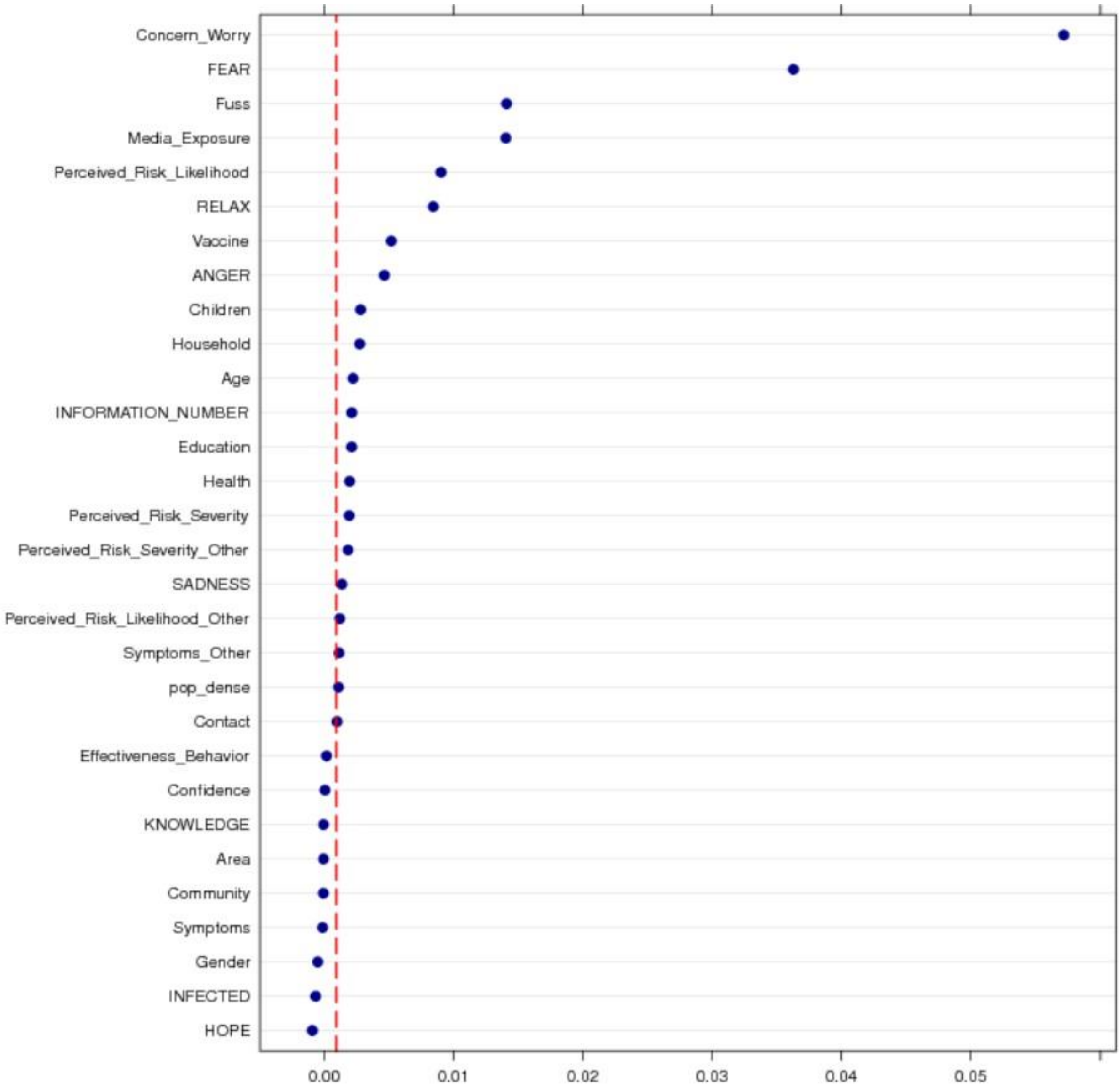

Figure S10. Permutation variable importance on hygiene behaviour (outcome; MTry = 6, seed = 2. ntree = 1000).

Table S3 *Regression results using hygiene behavior as the criterion.*

Predictor	*B*	*b* 95% CI [LL, UL]	*beta*	*beta* 95% CI [LL, UL]	*r*	Fit
(Intercept)	2.94*	[2.23, 3.66]				
Concern/Worry	0.14*	[0.08, 0.20]	0.10	[0.06, 0.15]	.25	
Fear	0.12*	[0.06, 0.18]	0.10	[0.05, 0.14]	.23	
Media Exposure	0.15*	[0.09, 0.21]	0.09	[0.06, 0.12]	.17	
Fuss	-0.11*	[-0.15, -0.07]	-0.10	[-0.13, -0.06]	-.20	
Perceived Risk (Likelihood	0.00	[0.00, 0.00]	0.04	[0.00, 0.07]	.12	

)						
Relaxation	-0.06	[-0.10, -0.01]	-0.05	[-0.09, -0.01]	-.19	
Anger	0.01	[-0.03, 0.05]	0.01	[-0.02, 0.05]	.07	
Vaccine	0.12	[0.03, 0.21]	0.04	[0.01, 0.08]	.07	
Amount of Media Sources	0.02	[-0.01, 0.06]	0.02	[-0.01, 0.06]	.05	
# of Children	-0.03	[-0.09, 0.02]	-0.03	[-0.08, 0.02]	.01	
Household Size	0.06	[0.02, 0.10]	0.06	[0.02, 0.11]	.05	
Sadness	-0.03	[-0.08, 0.02]	-0.03	[-0.07, 0.02]	.11	
Age	0.00	[-0.02, 0.03]	0.01	[-0.04, 0.05]	-.02	
Population Density (Municipality)	0.00	[-0.00, 0.00]	0.02	[-0.01, 0.06]	.04	
Symptom Close Others	0.03	[-0.05, 0.12]	0.01	[-0.02, 0.05]	.06	
Perceived Risk Close Others (Severity)	0.01	[-0.02, 0.04]	0.01	[-0.02, 0.05]	.08	
Education Level	0.02	[-0.01, 0.05]	0.03	[-0.01, 0.06]	.06	
Perceived Health	0.02	[-0.02, 0.07]	0.02	[-0.01, 0.06]	.00	
						R^2 = .106* 95% CI[.08,.12]

Note. A significant *b*-weight indicates the beta-weights are also significant. *b* represents unstandardized regression weights. *beta* indicates the standardized regression weights. *r* represents the zero-order correlation. *LL* and *UL* indicate the lower and upper limits of a confidence interval, respectively.
* indicates $p < .001$.

Results with different coding of Health-Protective Behavior.

In the original analyses, health-protective behaviour is coded by summing responses on whether participants engaged in the specific behaviour (yes = 1) or whether they did not (no = 0). Similarly, the answer alternatives ‘unsure’ and ‘not applicable’ were coded as 0. It is possible that some effects might be biased due to the fact that some options might not be applicable to some respondents (e.g. because they don’t have children, don’t work etc.). Thus, we recoded the health-protective behaviour

sum score and repeated all analyses using this variable. In this version we coded yes again with 1, and not applicable and unsure with 0. However, this time respondents choosing no were coded with -1. The score therefore ranged from -22 to 22 (*M* = 10.23, *SD* = 5.10).

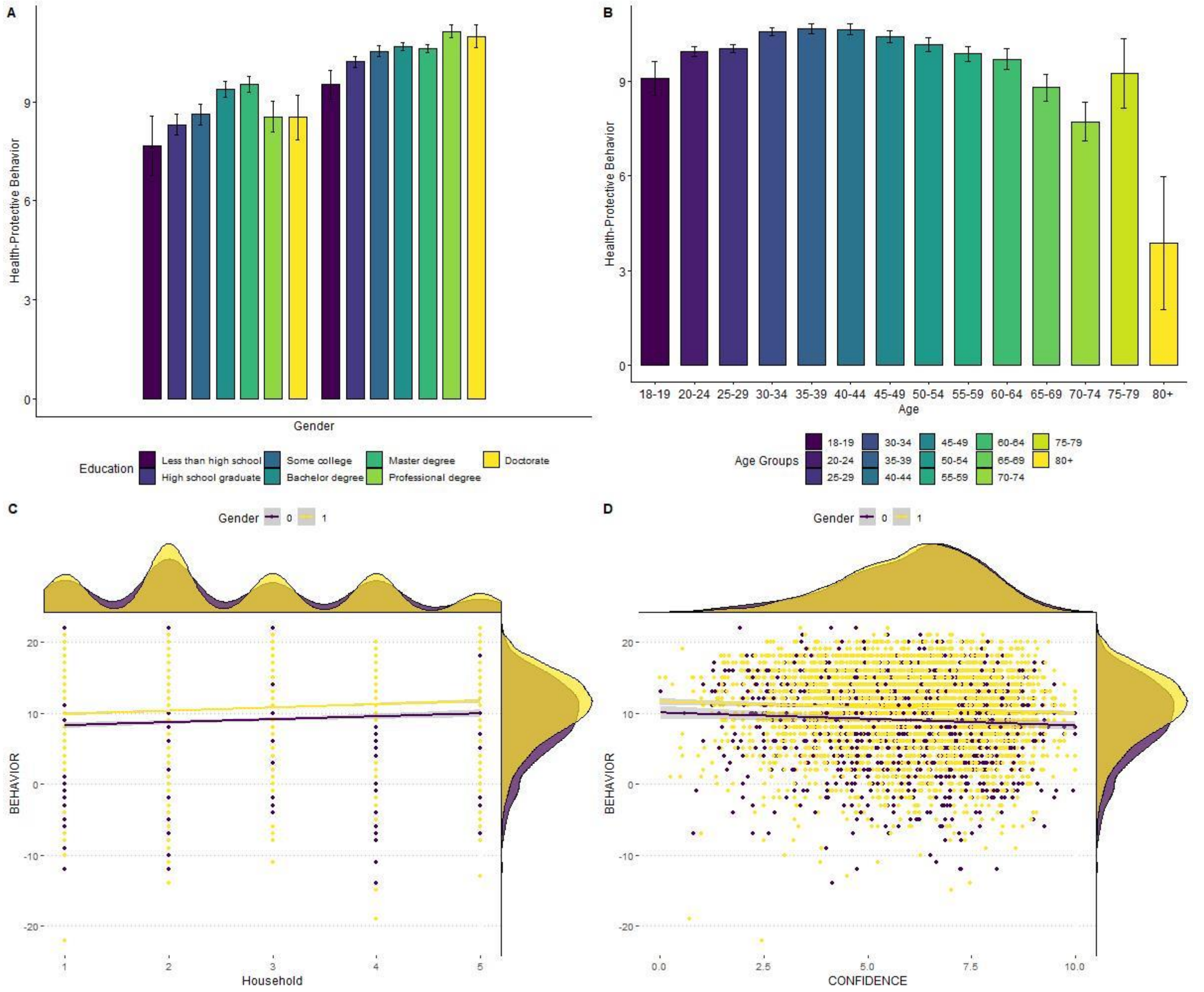

Figure S11. A. Health-protective behavior by gender and education level. B. Health-protective behavior by age group. C. Association of health-protective behavior and household size separately for gender. D. Association between health-protective behavior and confidence in authorities separately for gender.

Table S4 *Regression results using health-protective behavior as the criterion.*

Predictor	*b*	*b* 95% CI [LL, UL]	*beta*	*beta* 95% CI [LL, UL]	*r*	Fit
(Intercept)	4.47*	[2.88, 6.07]				
Gender	1.64*	[1.38, 1.90]	0.13	[0.11, 0.16]	.14	

Age	-0.04	[-0.09, 0.00]	-0.02	[-0.04, 0.00]	-.03	
Education Level	0.18*	[0.10, 0.26]	0.05	[0.03, 0.07]	.05	
Household Size	0.44*	[0.35, 0.53]	0.11	[0.09, 0.13]	.11	
						R^2 = .033*<br>95% CI[.03,.04]
(Intercept)	11.28*	[10.85, 11.70]				
Confidence in Authorities	-0.17*	[-0.24, -0.11]	-0.06	[-0.08, -0.03]	-.06	
						R^2 = .003<br>95% CI[.00,.01]

Note. A significant *b*-weight indicates the beta-weights are also significant. *b* represents unstandardized regression weights. *beta* indicates the standardized regression weights. *r* represents the zero-order correlation. *LL* and *UL* indicate the lower and upper limits of a confidence interval, respectively. Gender is dummy coded: male = 0, female = 1. *indicates *p* < .001.

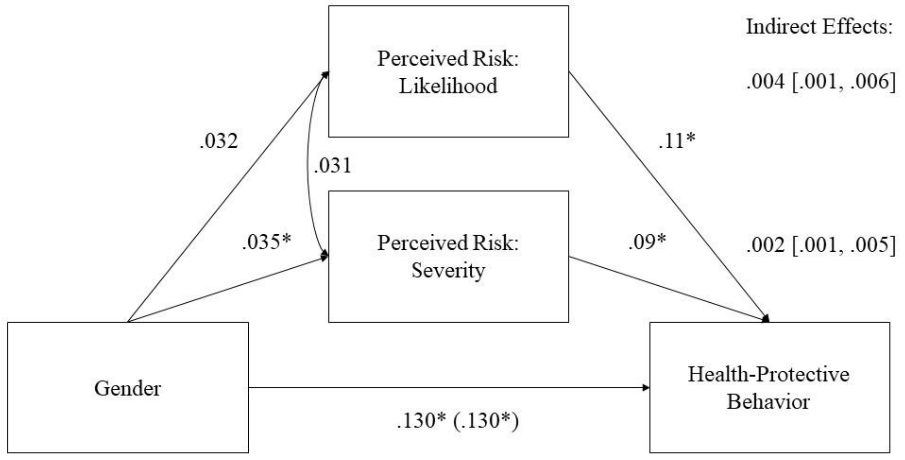

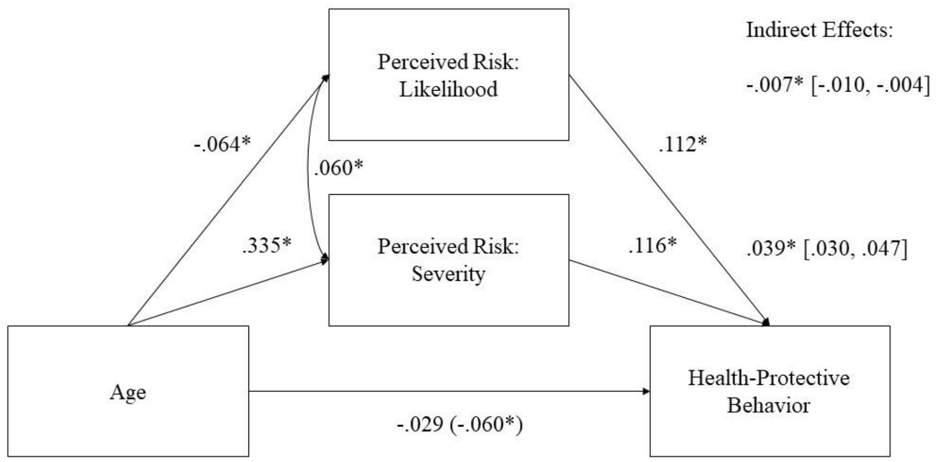

Indirect Effects:
-.007* [-.010, -.004]
Perceived Risk: Likelihood
-.064*
.060*
.112*
Perceived Risk: Severity
.335*
.116*
.039* [.030, .047]
Age
Health-Protective Behavior
-.029 (-.060*)

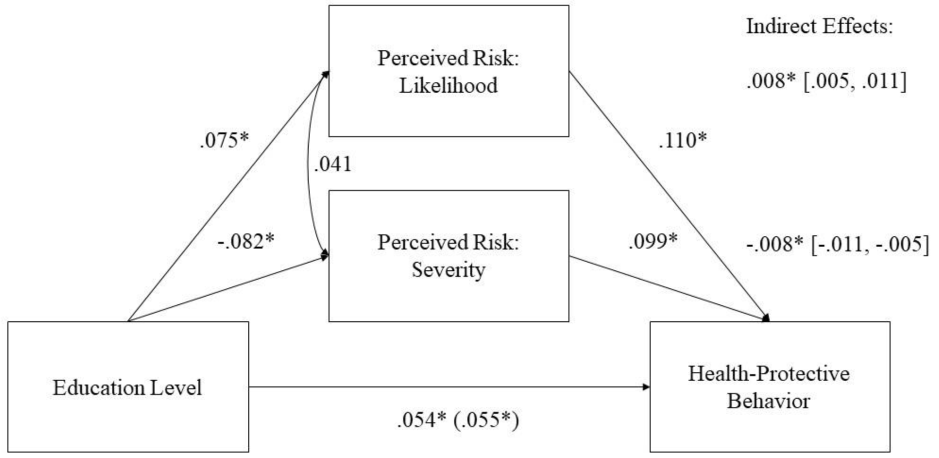

Indirect Effects:
.008* [.005, .011]
Perceived Risk: Likelihood
.075*
.041
.110*
Perceived Risk: Severity
-.082*
.099*
-.008* [-.011, -.005]
Education Level
Health-Protective Behavior
.054* (.055*)

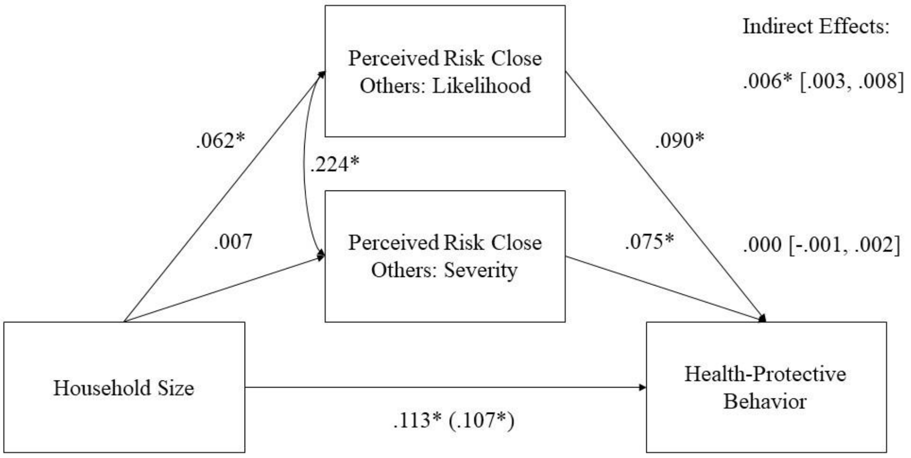

Figure S12. Mediation diagrams using gender (A; 0 = male, 1 = female), age (B), education level (C), and household size (D) as predictors, perceived risk likelihood and severity (A,B,C) or perceived risk likelihood and severity close others (D) as mediators, and health-protective behavior as the outcome. Estimates represent standardized estimates, confidence intervals are 95% bootstrap CIs. *indicates <.001.

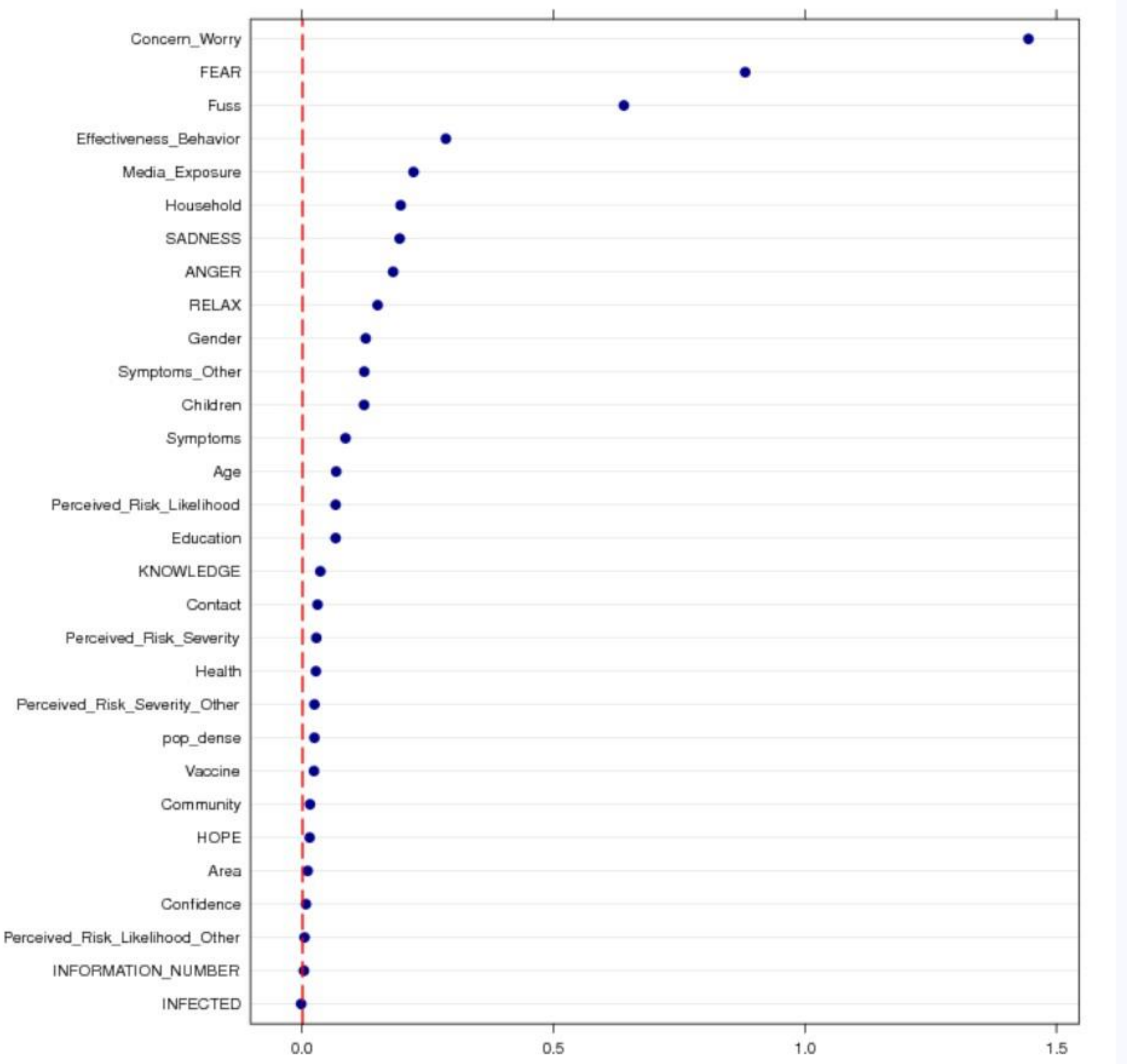

Figure S13. Permutation variable importance of predictors of health-protective behavior (seed 1, MTry = 5, ntree = 1000). Variables exceeding the red line are unlikely to result from random noise.

Table S5. *Regression results using health-protective behavior as the criterion.*

Predictor	*b*	*b* 95% CI [LL, UL]	*beta*	*beta* 95% CI [LL, UL]	*r*	Fit
(Intercept)	-0.44	[-4.31, 3.44]				
Concern/Worry	0.51*	[0.22, 0.79]	0.09	[0.04, 0.14]	.29	
Fear	0.43	[0.16, 0.70]	0.09	[0.03, 0.14]	.27	
Fuss	-0.61*	[-0.80, -0.42]	-0.13	[-0.17, -0.09]	-.26	
Effectiveness Behavior	0.03*	[0.02, 0.05]	0.11	[0.07, 0.15]	.11	
Media Exposure	0.57*	[0.31, 0.84]	0.08	[0.04, 0.12]	.19	
Household Size	0.33	[0.12, 0.53]	0.08	[0.03, 0.13]	.13	

Sadness	0.01	[-0.21, 0.23]	0.00	[-0.04, 0.05]	.15
Anger	0.07	[-0.11, 0.25]	0.02	[-0.03, 0.06]	.11
Relaxation	-0.27	[-0.47, -0.07]	-0.06	[-0.10, -0.02]	-.23
Gender	0.80*	[0.35, 1.26]	0.07	[0.03, 0.10]	.14
Symptoms Other	0.26	[-0.18, 0.69]	0.02	[-0.02, 0.07]	.12
# Children	0.38	[0.10, 0.66]	0.08	[0.02, 0.14]	.08
Symptoms	0.55	[0.08, 1.01]	0.05	[0.01, 0.09]	.13
Age	-0.16	[-0.27, -0.05]	-0.08	[-0.13, -0.03]	-.06
Perceived Risk Likelihood	0.01	[-0.00, 0.01]	0.02	[-0.02, 0.06]	.13
Education Level	0.11	[-0.03, 0.25]	0.03	[-0.01, 0.07]	.05
Knowledge	-0.08	[-0.15, -0.01]	-0.04	[-0.08, -0.01]	.01
Contact	0.68	[0.15, 1.21]	0.05	[0.01, 0.09]	.08
Perceived Risk Severity	0.14	[-0.14, 0.42]	0.02	[-0.02, 0.06]	.07
Perceived Health	0.02	[-0.20, 0.23]	0.00	[-0.04, 0.05]	-.03
Perceived Risk Close Others: Severity	0.17	[0.03, 0.32]	0.04	[0.01, 0.08]	.12
Population Density Municipality Level	0.00	[-0.00, 0.00]	0.02	[-0.03, 0.07]	.03
Vaccine	0.30	[-0.12, 0.73]	0.03	[-0.01, 0.06]	.05
Community Type	-0.14	[-0.37, 0.10]	-0.03	[-0.08, 0.02]	-.03

R^2 = .185*
95% CI[.15,.20]

Note. A significant *b*-weight indicates the beta-weights are also significant. *b* represents unstandardized regression weights. *beta* indicates the standardized regression weights. *r* represents the zero-order correlation. *LL* and *UL* indicate the lower and upper limits of a confidence interval, respectively. * indicates $p < .001$.

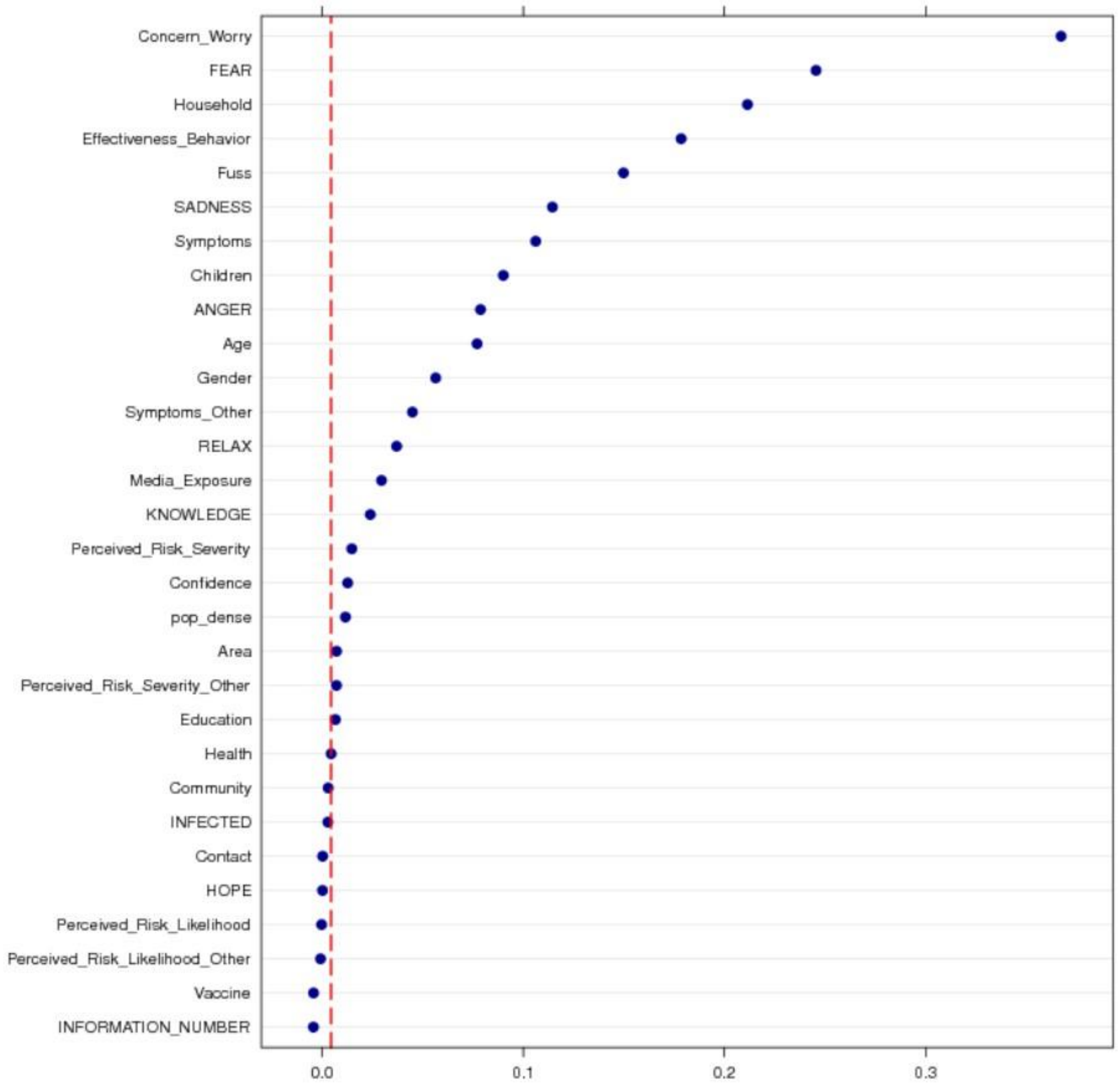

Figure S14. Permutation variable importance of predictors of physical distancing (seed 1, MTry = 5, ntree = 1000). Variables exceeding the red line are unlikely to result from random noise.

Table S6. *Regression results using physical distancing as the criterion.*

Predictor	*b*	*b* 95% CI [LL, UL]	*beta*	*beta* 95% CI [LL, UL]	*R*	Fit
(Intercept)	3.46*	[1.69, 5.23]				
Concern/Worry	0.22	[0.03, 0.41]	0.06	[0.01, 0.10]	.20	
Fear	0.26	[0.09, 0.44]	0.08	[0.03, 0.13]	.20	
Household Size	0.31*	[0.17, 0.44]	0.11	[0.06, 0.16]	.16	
Effectiveness Behavior	0.02*	[0.01, 0.03]	0.11	[0.07, 0.14]	.10	
Fuss	-0.39*	[-0.51, -0.27]	-0.12	[-0.16, -0.08]	-.20	
Sadness	-0.03	[-0.17, 0.12]	-0.01	[-0.05,	.10	

				0.04]		
Symptoms	0.57*	[0.28, 0.85]	0.08	[0.04, 0.11]	.13	
# Children	0.29	[0.12, 0.47]	0.09	[0.04, 0.15]	.09	
Anger	0.03	[-0.09, 0.15]	0.01	[-0.03, 0.05]	.08	
Age	-0.15*	[-0.22, -0.08]	-0.11	[-0.16, -0.06]	-.08	
Gender	0.54*	[0.24, 0.84]	0.06	[0.03, 0.10]	.12	
Symptom Close Others	0.26	[-0.02, 0.53]	0.04	[-0.00, 0.07]	.11	
Relaxation	-0.06	[-0.19, 0.07]	-0.02	[-0.06, 0.02]	-.15	
Media Exposure	0.20	[0.02, 0.38]	0.04	[0.01, 0.08]	.11	
Knowledge	-0.05	[-0.09, -0.01]	-0.04	[-0.08, -0.00]	-.01	
Perceived Risk: Severity	0.11	[-0.05, 0.28]	0.03	[-0.01, 0.06]	.05	
Confidence in Authorities	-0.05	[-0.13, 0.03]	-0.02	[-0.06, 0.02]	-.05	
Population Density Municipality Level	0.00	[0.00, 0.00]	0.04	[0.01, 0.08]	.01	
						R^2 = .129*<br>95% CI[.10,.15]

Note. A significant *b*-weight indicates the beta-weights are also significant. *b* represents unstandardized regression weights. *beta* indicates the standardized regression weights. *r* represents the zero-order correlation. *LL* and *UL* indicate the lower and upper limits of a confidence interval, respectively. * indicates $p < .001$.

Table S7. *Regression results using hygiene behavior as the criterion.*

Predictor	*b*	*b*<br>95% CI<br>[LL, UL]	*beta*	*beta*<br>95% CI<br>[LL, UL]	*r*	Fit
(Intercept)	-1.11	[-2.38, 0.17]				
Concern	0.26**	[0.15, 0.37]	0.10	[0.06, 0.14]	.26	
Fear	0.26**	[0.15, 0.36]	0.11	[0.07, 0.15]	.24	
Media Exposure	0.30**	[0.20, 0.40]	0.10	[0.06, 0.13]	.18	
Fuss	-0.20**	[-0.27, -0.13]	-0.09	[-0.13, -0.06]	-.20	
Relaxation	-0.08*	[-0.16, -0.01]	-0.04	[-0.08, -0.00]	-.19	

Vaccine	0.24**	[0.08, 0.40]	0.05	[0.02, 0.08]	.08
Perceived Risk: Likelihood	0.00	[-0.00, 0.01]	0.02	[-0.02, 0.06]	.12
Anger	0.06	[-0.01, 0.13]	0.03	[-0.00, 0.06]	.09
Perceived Risk: Severity	0.04	[-0.06, 0.14]	0.01	[-0.02, 0.05]	.07
Education Level	0.06*	[0.00, 0.11]	0.03	[0.00, 0.06]	.06
Perceived Risk Close Other: Likelihood	0.00	[-0.00, 0.01]	0.03	[-0.01, 0.07]	.12
Population Density Municipality	0.00	[-0.00, 0.00]	0.01	[-0.02, 0.05]	.04
Sadness	-0.05	[-0.13, 0.04]	-0.02	[-0.06, 0.02]	.12
Perceived Health	0.09*	[0.01, 0.17]	0.04	[0.00, 0.07]	.00

R^2 = .108*
95% CI[.09,.12]

Note. A significant *b*-weight indicates the beta-weights are also significant. *b* represents unstandardized regression weights. *beta* indicates the standardized regression weights. *r* represents the zero-order correlation. *LL* and *UL* indicate the lower and upper limits of a confidence interval, respectively. * indicates $p < .001$.

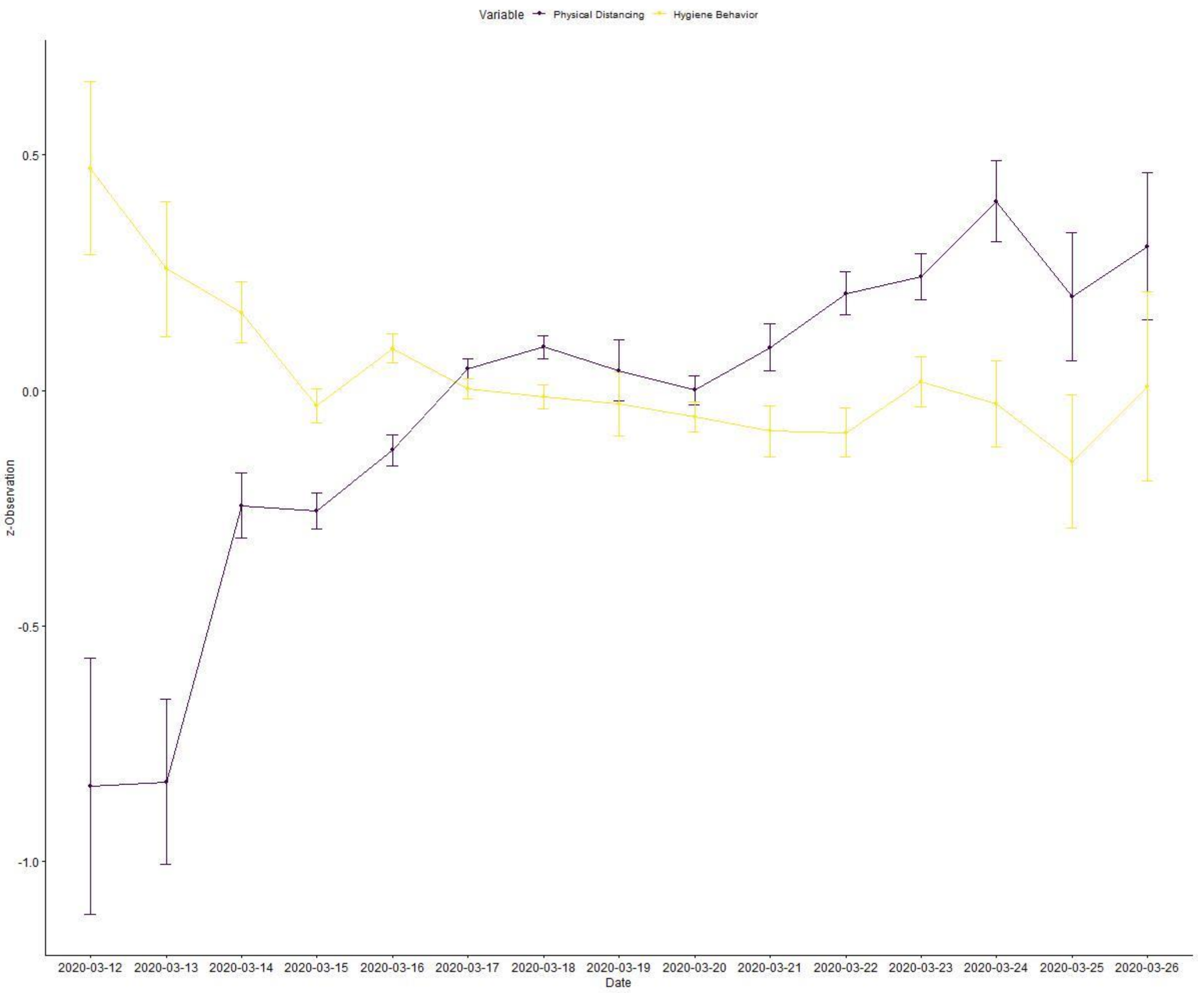

Figure S15. Time series of behavior. All variables are z-standardized to ease comparisons. Error bars represent standard errors.

In sum, results were quite similar to the alternative coding used in the main manuscript. The age effect was slightly reduced, although it also occurred in the context of social distancing behaviors. Otherwise, findings were highly comparable.

Results of Confidence in Chinese government.

Finally, we explored the association between confidence whether the Chinese government provides accurate and full information and health-protective behavior.

On average, confidence whether the Chinese government provided accurate information was somewhat lower than the midpoint of the scale (*M* = 4.34, *SD* = 2.26). Running a linear regression model, we found no significant effect of confidence in the Chinese government on health-protective behavior, *B* = .007, *SE* = .016, *p* = .689, or specifically on social distancing, *B* = -.004, *SE* = .013, *p* = .76, or hygiene behavior, *B* = .008, *SE* = .006, *p* = .155.
